# Ineffectiveness of hemoadsorption in large animals with abdominal sepsis: a randomized controlled porcine study

**DOI:** 10.1186/s40635-024-00622-x

**Published:** 2024-04-18

**Authors:** Vaclav Tegl, Jan Horak, Lukas Nalos, Michala Horakova, Milan Stengl, Martin Matejovic, Jan Benes

**Affiliations:** 1 Laboratory of Experimental Intensive Care Medicine, Biomedical Center, Faculty of Medicine in Pilsen, Charles University, Alej Svobody 1655/76, 323 00 Pilsen, Czech Republic; 2 Department of Anesthesiology, Resuscitation and Intensive Care, Faculty of Medicine in Pilsen, Charles University, Pilsen, Czech Republic; 3 Department of Anesthesiology, Resuscitation and Intensive Care, Faculty Hospital in Pilsen, Pilsen, Czech Republic; 4 Department of Internal Medicine I, Faculty of Medicine in Pilsen, Charles University, Pilsen, Czech Republic; 5 Department of Physiology, Faculty of Medicine in Pilsen, Charles University, Pilsen, Czech Republic; 6 Laboratory of Experimental Cardiology, Biomedical Center, Faculty of Medicine in Pilsen, Charles University, Pilsen, Czech Republic

**Keywords:** Sepsis, Septic shock, Adsorption, Investigational therapies, Hemoperfusion

## Abstract

**Objectives:**

The use of hemoadsorption (HA) has become popular in the treatment of vasoplegic states associated with massive cytokine release, including septic shock. However, this approach does not seem to be based on robust evidence, and it does not follow international guidelines. To understand the pathophysiological rationale and timing of HA, we conducted a large animal septic shock experiment.

**Design:**

Prospective randomized large-animal peritoneal septic shock experiment.

**Setting:**

Laboratory investigation.

**Subjects:**

Twenty-six anesthetized, mechanically ventilated, and instrumented pigs randomly assigned into (1) sham-operated group with HA (SHAM, *n* = 5); (2) sepsis animals without HA (SEPSIS, *n* = 5); (3) sepsis group with HA at norepinephrine initiation (EARLY, *n* = 8); and (4) sepsis group with HA initiated at norepinephrine rate reaching 0.5 μg/kg/min (LATE, n = *8*).

**Interventions:**

Peritoneal sepsis was induced by cultivated autologous feces inoculation. A CytoSorb cartridge (200 g) with a blood flow rate of 200 mL/min and heparin anticoagulation was used to perform HA. The animals received sedation and intensive organ support up to 48 h or until they experienced cardiovascular collapse.

**Measurements and main results:**

Systemic hemodynamics, multiple-organ functions, and immune-inflammatory response were measured at predefined periods. The HA treatment was not associated with any measurable benefit in terms of systemic hemodynamics and organ support. The systemic inflammatory markers were unaffected by any of the treatment timings. In contrast, the HA resulted in higher vasopressor load and decreased 36-h survival (5 animals in SHAM (100%), 4 (80%) in SEPSIS, 4 (57%) in EARLY, and 2 (25%) in LATE; *p* = 0.041). The HA exposure in healthy animals was associated with hemodynamic deterioration, systemic inflammatory response, and cytopenia.

**Conclusions:**

In this large-animal-controlled fulminant sepsis study, the HA was unable to counteract the disease progression in the early or advanced septic shock phase. However, findings from the HA-exposed sham animals suggest potential safety concerns.

**Supplementary Information:**

The online version contains supplementary material available at 10.1186/s40635-024-00622-x.

## Keypoints

Question: To understand hemoadsorption using CytoSorb device pathophysiological rationale and timing on disease progression in septic subjects.

Findings: In this large-animal-controlled fulminant sepsis study, the CytoSorb hemoadsorption did not counteract the disease progression in the early or advanced septic shock phase. However, findings from the hemoadsorption-exposed sham animals suggest potential safety concerns.

Meaning: Considering the limited evidence, the clinical use of CytoSorb for hemoadsorption in septic patients should be limited to well-designed interventional studies.

**Supplementary Information:**

The online version contains supplementary material available at 10.1186/s40635-024-00622-x.

## Introduction

Despite decades of attempts, the mortality rate of septic shock patients is as high as 40% [1]. Given the extreme complexity of sepsis pathogenesis, the paradigm “one disease, one drug” is flawed. Therapeutic strategies aimed at modulating complex dysregulated immune-inflammatory host response may have both scientific and clinical relevance. In this context, non-selective hemoadsorption (HA) extracorporeal methods theoretically capable of attenuating the host response represent attractive pathways. CytoSorb (CS) (CytoSorbents Europe GmbH, Germany) is a cartridge containing biocompatible polystyrene divinylbenzene copolymer beads coated with polyvinylpyrrolidone. When incorporated into an extracorporeal circuit, it may adsorb molecules of mid-molecular weight (5–55 kDa).

Although the use of HA is not supported by the current Sepsis Surviving guidelines [2], several case series, especially involving CS, show promising signals [3, 4]. However, none of the few published randomized trials using CS have shown significant beneficial outcomes [5–9] and a few authors even raised safety concerns [10]. Even though several multicenter randomized trials are ongoing [11, 12], the recently published meta-analyses concluded that HA effectiveness and safety are not supported by the current data [13, 14]. Recent International expert consensus for pre-clinical sepsis studies recommends obtaining compelling preclinical evidence from large-animal models before proceeding to clinical trials [15]. Because of this, the application of clinical HA in sepsis is based on insufficient evidence and should be carefully considered [16].

With this background, we aimed to examine the effects of HA on systemic hemodynamics, vasopressor requirement, energy metabolism, organ function, systemic inflammatory response, and survival time in a clinically relevant porcine model of peritonitis-induced progressive sepsis. In addition, to address the effect of timing, the study was designed to compare early and delayed HA initiation.

## Materials and methods

The trial was performed in the animal experimental intensive care facility at the Biomedical Center, Faculty of Medicine in Pilsen, Charles University, Czech Republic. Animal handling was in accordance with the European Directive for the Protection of Vertebrate Animals Used for Experimental and Other Scientific Purposes (86/609/EU) and approved by the Committee for Experiments on Animals of the Charles University Faculty of Medicine in Pilsen and by the Ministry of Education, Youth and Sports of the Czech Republic (MSMT-18726/2019-3 issued 28.6.2019). Twenty-six domestic pigs (*Sus scrofa f. domestica—*15 barrows, 11 sows) with similar weights [median 50.8 kg (interquartile range 47–57)] were used. The animals were obtained from local breeders as established in our laboratory; the usual minimum 2-week quarantine took place in our facility with daily veterinary check-ups. During this period, the animals were fed dry granules with unlimited access to water, but the animals underwent solid fasting for 12 h just before the surgery. On the morning of the experiment, randomization into four groups using sealed envelopes was performed before the commencement of the experiment:

1. sepsis group with standard supportive care (SEPSIS, *n* = 5),
2. sepsis group with HA initiated at the moment of norepinephrine indication (EARLY, *n* = 8),
3. sepsis group with HA initiation with a norepinephrine dose of 0.5 μg/kg/min (LATE, *n* = 8)
4. the sham-operated group with HA (SHAM, *n* = 5).

Because of the interventional nature of the study, concealment was not possible for clinical evaluation, but independent personnel performed all other measurements (i.e., biochemical and immunological) and statistical analysis.

### Anesthesia and instrumentation

The anesthesia and instrumentation protocols were similar to those previously described [17, 18]. The pigs received intramuscular tiletamine (2.2 mg/kg), zolazepam (2.2 mg/kg), and xylazine (2.2 mg/kg). Anesthesia was induced and maintained using intravenous propofol (induction: 1–2 mg/kg; maintenance: 1–4 mg/kg/h) and fentanyl infusions (5–10 µg/kg/h). Muscle paralysis using rocuronium (0.6 mg/kg) facilitated tracheal intubation and maintenance (0.15 mg/kg/h). Lung-protective mechanical ventilation was maintained throughout the experiment (FiO_2_ 0.3, PEEP 8 cmH_2_O, tidal volume 8 mL/kg) with adaptations to reach normoxemia (SpO_2_ 92–98%) and normocapnia (ETCO_2_ 4–5 kPa). Ringerfundin solution (B. Braun Melsungen AG, Germany) was infused at a rate of 7 mL/kg/h, and normoglycemia (blood glucose 4.5–7 mmol/L) was reached using 10% glucose (1–4 mL/kg/h).

All pigs received a thermistor-tipped femoral artery catheter, triple lumen central venous catheter, and pulmonary artery catheter for hemodynamic monitoring and blood sampling. In the HA-treated groups, a 16F dialysis cannula was inserted into the femoral vein. Silicone drains into the Morison and Douglas anatomical spaces facilitated fecal inoculation. In all animals, a 6 h recovery period was allowed before taking the first set of measurements and sepsis induction. Neither antimicrobial prophylaxis/treatment nor a source control was provided. To study the potential of the HA without any other synergistic effects, only life-sustaining treatments (ventilator and hemodynamic support) were performed.

### Experimental protocol

Autologous feces (2 g/kg, cultivated 4 h in 200 mL of saline at 38 °C) were instilled into the peritoneal cavity to induce peritonitis in all septic groups. Repeated crystalloid boluses (Ringerfundin, 5 mL/kg) were used to optimize the fluid loading based on the stroke volume variation (target 15%) and previous positive cardiac output reaction (an increase of more than 10%). Norepinephrine infusion was initiated in case of fluid-loading irresponsive hypotension (MAP ≤ 65 mmHg) and titrated to reach the MAP ≥ 65 mmHg.

The HA trigger depended on randomization: in the EARLY group, the trigger was the moment of fluid-loading irreversible shock (norepinephrine initiation). In the SHAM group, the timing was dictated by the previous animal from the EARLY group. The trigger in the LATE group was the norepinephrine infusion rate of 0.5 μg/kg/min. An extracorporeal hemoadsorption circuit coupled with one 200 g CytoSorb cartridge was used. The target blood flow rate was 200 mL/min. Systemic heparin anticoagulation was used with an activated clotting time target range of 300–400 s. The CS filter was not changed over the run of the experiment and no other extracorporeal blood purification method was used.

### Measurements

**Fig. 1:**
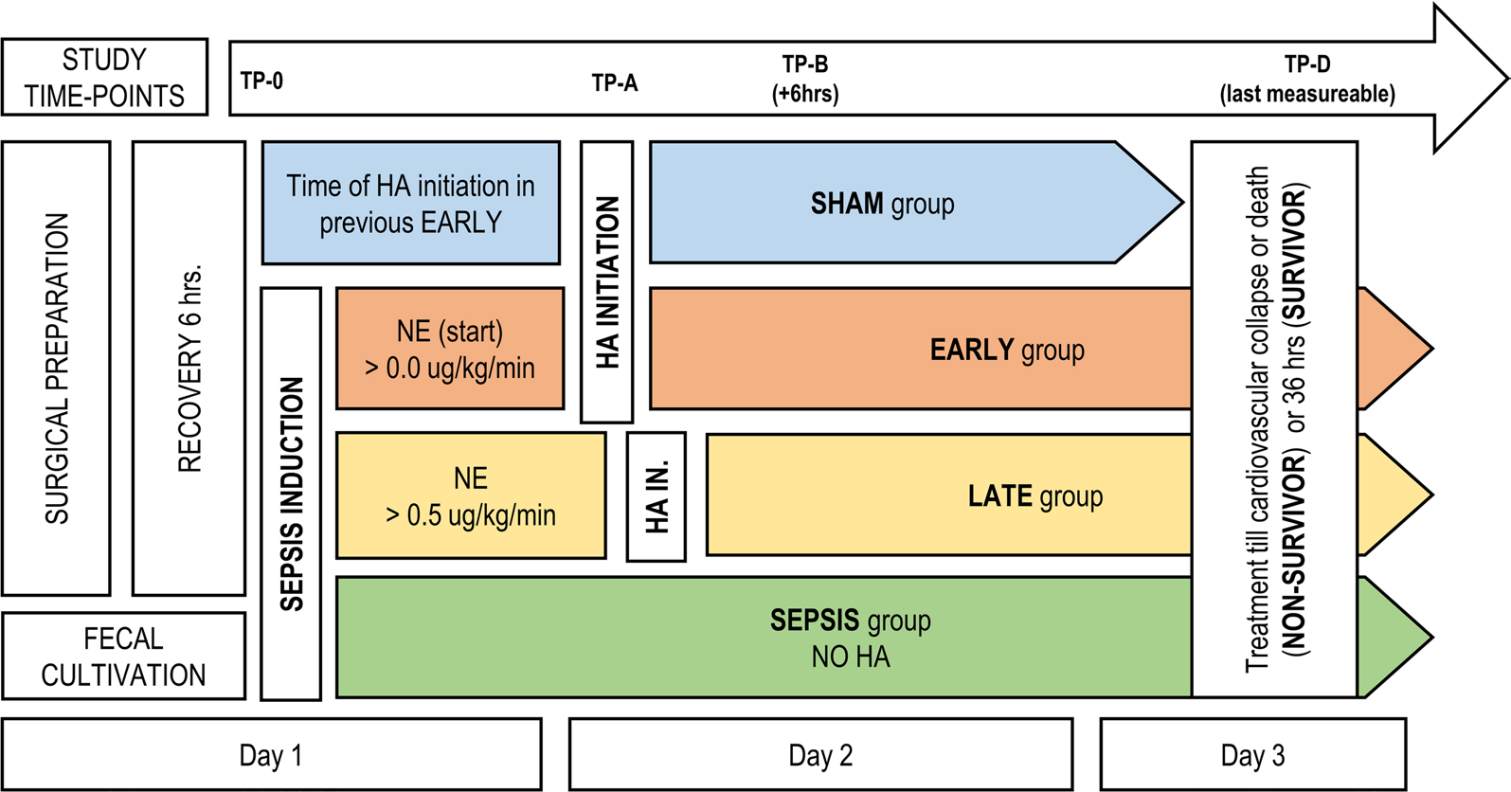
Scheme of the experimental protocol. *HA* hemoadsorption, *NE* norepinephrine (noradrenaline), *TP-0* start of the experiment/fecal inoculation, *TP-A* start of the HA or defined equivalent, *TP-B* six hours of the HA treatment, *TP-D* last measureable value

Throughout the experiment, we continuously monitored the standard set of ICU variables (pulse oximetry, continuous arterial and pulmonary pressures, ECG lead II, temperature, respiratory mechanics, capnography, and pulse contour-derived advanced hemodynamic parameters). Besides, intermittent pulmonary artery catheter derived cardiac output, transpulmonary termodilution, and bedside blood-gas analyses were performed at dedicated timepoints and in cases of clinical needs. The animals were continuously monitored throughout the experiment by an experienced intensivist. Analyses of the outcomes were based on four time-points (Fig. 1): TP-0 (baseline measurement), TP-A (the HA initiation or equivalent time-point), TP-B (6 h thereafter) and TP-D (the last measurable set before the death of the animal). Animals that survived beyond 36 h (30 h after TP-0) were marked as “survivors”. Those in the SHAM group were euthanized at this point, but animals in the septic group received further support until cardiovascular collapse occurred.

The primary endpoint of the study was the impact of the HA treatment itself and its timing on the survival of the animal, hemodynamic stability/vasoplegic shock progression served as the secondary endpoint.

### Statistical analysis

The results are represented as medians (interquartile range) or counts (percentage). No a priori sample size calculation was possible based on previous literature. Shapiro–Wilk test was used for normality testing. The groups were compared using ANOVA/Kruskall–Wallis with Tukey/Dunn post-hoc testing or unpaired T-test/Mann–Whitney tests. For comparison of temporal evolution, repeated measurement ANOVA/Friedmann or paired *T* test/Wilcoxon tests were used as appropriate. The Chi-square test was used for frequency comparison. Secondary analyses with joint EARLY and LATE groups (septic HA exposed animals: SEP-HA) and septic animals without HA (group SEPSIS) were performed. The effect of HA exposure on the sham-operated animals (SHAM) was compared to the historical healthy CONTROL from our most recent experiment of a similar design [18]—a reduction demanded by the ethics committee. The analysis was performed using MedCalc^®^ Statistical Software version 20.106 (MedCalc Software Ltd, Belgium). Values of *p* < 0.05 were considered significant.

## Results

In total, 26 animals were used for this experiment. During the recovery period, one EARLY group animal was excluded after randomization for technical issues (i.e., uncontrolled surgical bleeding). The study sample size consists of 5 sham-operated HA-exposed animals (SHAM), 5 septic controls without HA (SEPSIS), 7 septic animals with HA started with septic shock onset (EARLY) and 8 septic animals with HA started at refractory shock (LATE). No major differences exist in terms of gender or weight distribution between the groups (Table 1). Survival of the animals significantly differed between groups with and without HA treatment; especially the LATE group being significantly worse (Table 1).

**Table 1:** General characteristics of the experimental animals

	Sham	Sepsis	Early	Late	*p*
Gender (F/M)	3/2	3/2	4/3	4/4	0.9799
Weight (kg)	57 (48.5–59.5)	56 (48.5–58)	52 (49–54)	49 (44–51)	0.3353
Survival in 36 h of the experiment	5 (100%)	4 (80%)	4 (57%)	2 (25%)	**0.0407**
Time T0 to death (min)	1800 (1800–1800)	**2160 (1946–2325)#**	1920 (1747–2160)	**1703 (1440–1790)#**	**0.0469**
Time T0 to HA initiation (min)	1140 (900–1275)	N/A	1260 (1080–1395)	1200 (1035–1410)	0.6786
Length of HA treatment (min)	600 (480–900)	N/A	690 (525–769)*	375 (330–503)*	**0.0206**
Time T0 to NE (min)	970 (870–1215)	1200 (1143–1458)	1260 (1099–1398)	963 (763–1133)	0.0899
Time from NE initiation to 0.3 μg/kg/min (min)	210 (145–1013)*	80 (68–165)	30 (20–51)*	144 (73–378)	**0.0253**
Time from NE initiation to 0.5 μg/kg/min (min)	400 (299–1061)*	190 (174–246)	55 (41–66)*	205 (136–443)	**0.0117**
Time from NE initiation to 1.0 μg/kg/min (min)	590 (369–1099)	360 (248–490)	210 (130–390)	330 (203–550)	0.1976
Time from NE initiation to 3.0 μg/kg/min (min)	N/A	610 (440–940)	380 (278–607)	683 (393–1285)	0.3371
NE average speed (μg/kg/min)	0.9 (0.4–1.5)*	2.3 (2.1–2.7)	3.7 (3.0–4.5)*	3.0 (1.4–6.7)	**0.0137**
NE cumulative dose (μg/kg/h)	26.6 (8.2–49.0)	56.2 (46.4–62.5)	69.5 (61.3–98.2)	81.5 (34.4–138.3)	0.0906
Diuresis (mL/kg/h)	1.4 (1.2–1.5)*	0.9 (0.6–1.0)	0.9 (0.8–1.0)	0.7 (0.6–0.9)*	**0.0057**
Fluid intake (mL/kg/h)	9.7 (9.2–10.0)	10.3 (9.2–10.6)	10.1 (9.5–11.4)	10.3 (9.7–10.8)	0.2975
Fluid balance (mL/kg/h)	8.3 (7.6–8.7)	9.4 (8.3–9.7)	9.4 (8.6–10.5)	9.4 (9.1–10.1)	0.0565

Data are presented as number (proportion) or median (25th–75th quartile/interquartile range)

*F* female/sow, *M* male/boar, *NE* norepinephrine, *HA* hemoadsorption

*Statistical significance (*p* < 0.05) in ANOVA/Kruskall Wallis ANOVA on ranks test with intergroup comparison (bold indicates groups with a difference)

#Statistical significance (*p* < 0.05) in Kruskall Wallis ANOVA on ranks test within sepsis groups only (bold indicates groups with a difference)﻿

### Hemodynamic effects of HA in sepsis

**Fig. 2:**
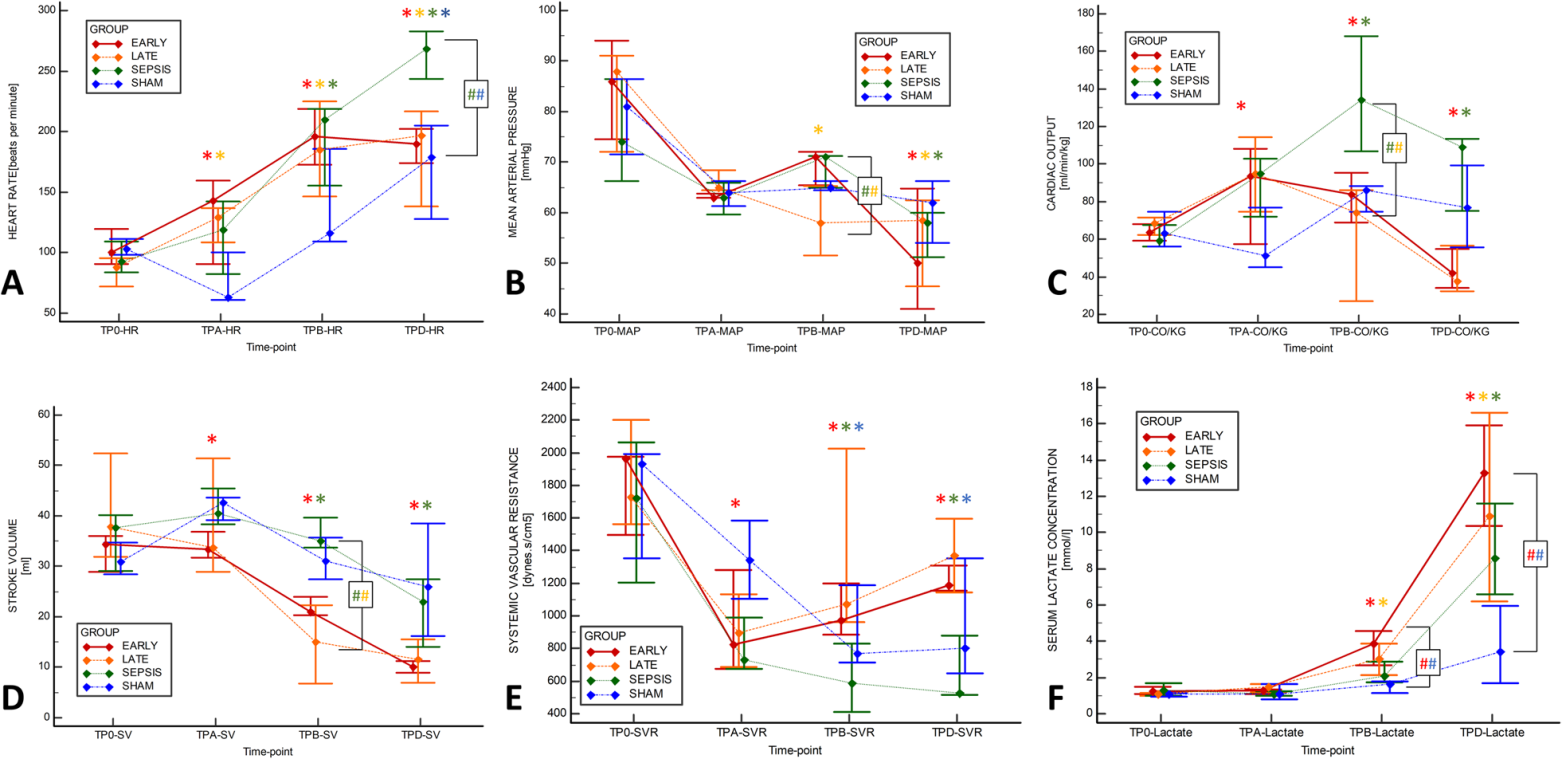
Evolution of major hemodynamic parameters and serum lactate levels. **A** Heart rate, **B** Mean arterial pressure, **C** Cardiac output per kg, **D** Stroke volume, **E** Systemic vascular resistance, **F** Lactate serum level. The graph displays the median (diamond) and 25–75% quartile range with connecting lines. *TP-0* start of the experiment/fecal inoculation, *TP-A* start of the HA or defined equivalent, *TP-B* 6 h of the HA treatment, *TP-D* last measureable value. Asterisk (*) in adequate color marks significance vs. baseline (RM ANOVA: Friedman test); Hashtag (#) in corresponding colors marks significant intergroup difference in given time-point (Kruskal Wallis ANOVA on ranks)

**Fig. 3:**
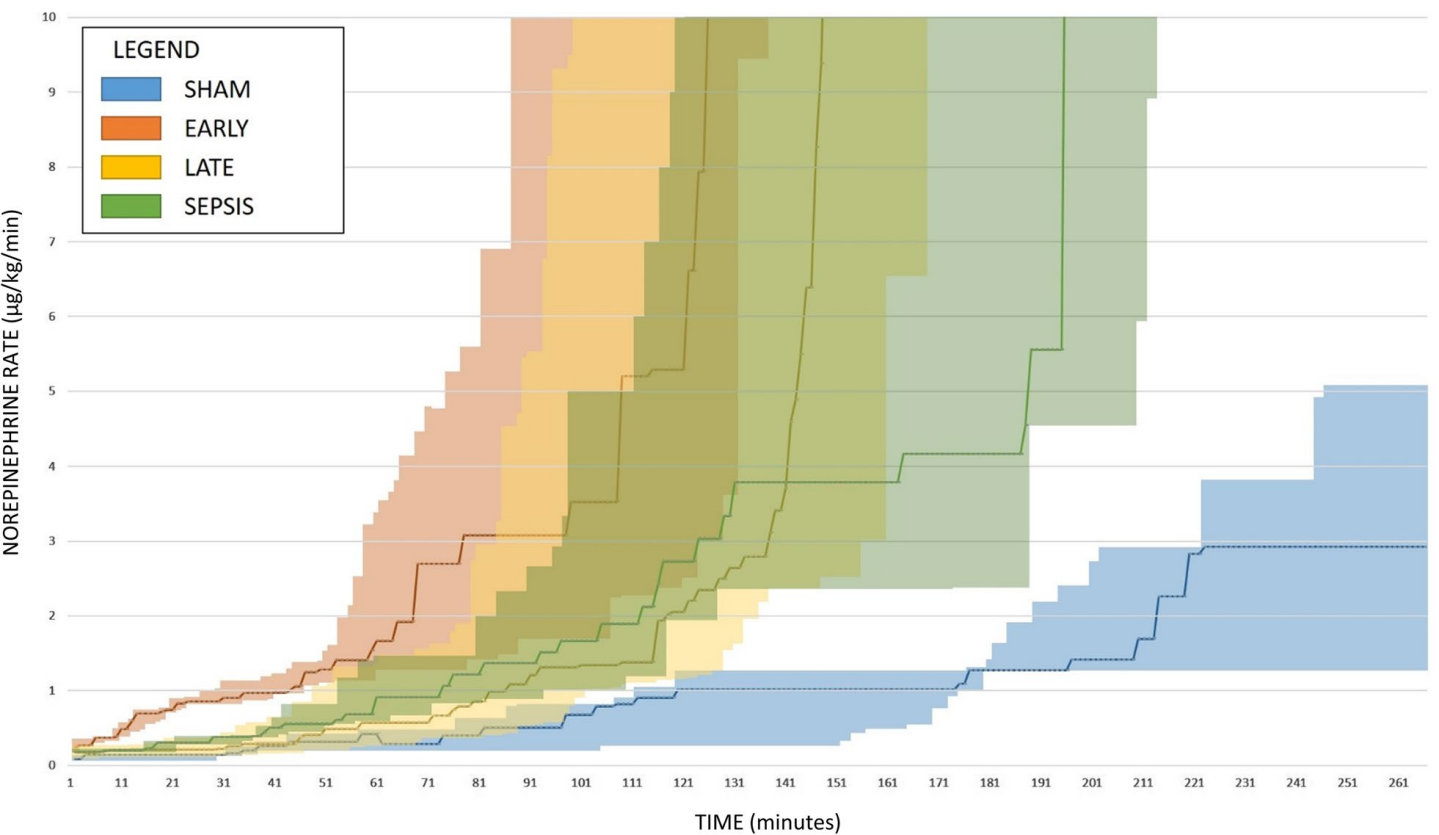
Norepinephrine infusion rate evolution. Starting point is the moment of the first norepinephrine infusion. Line corresponds to the median value for the group in the dedicated time; shaded area corresponds to the 25–75 quartile values

**Fig. 4:**
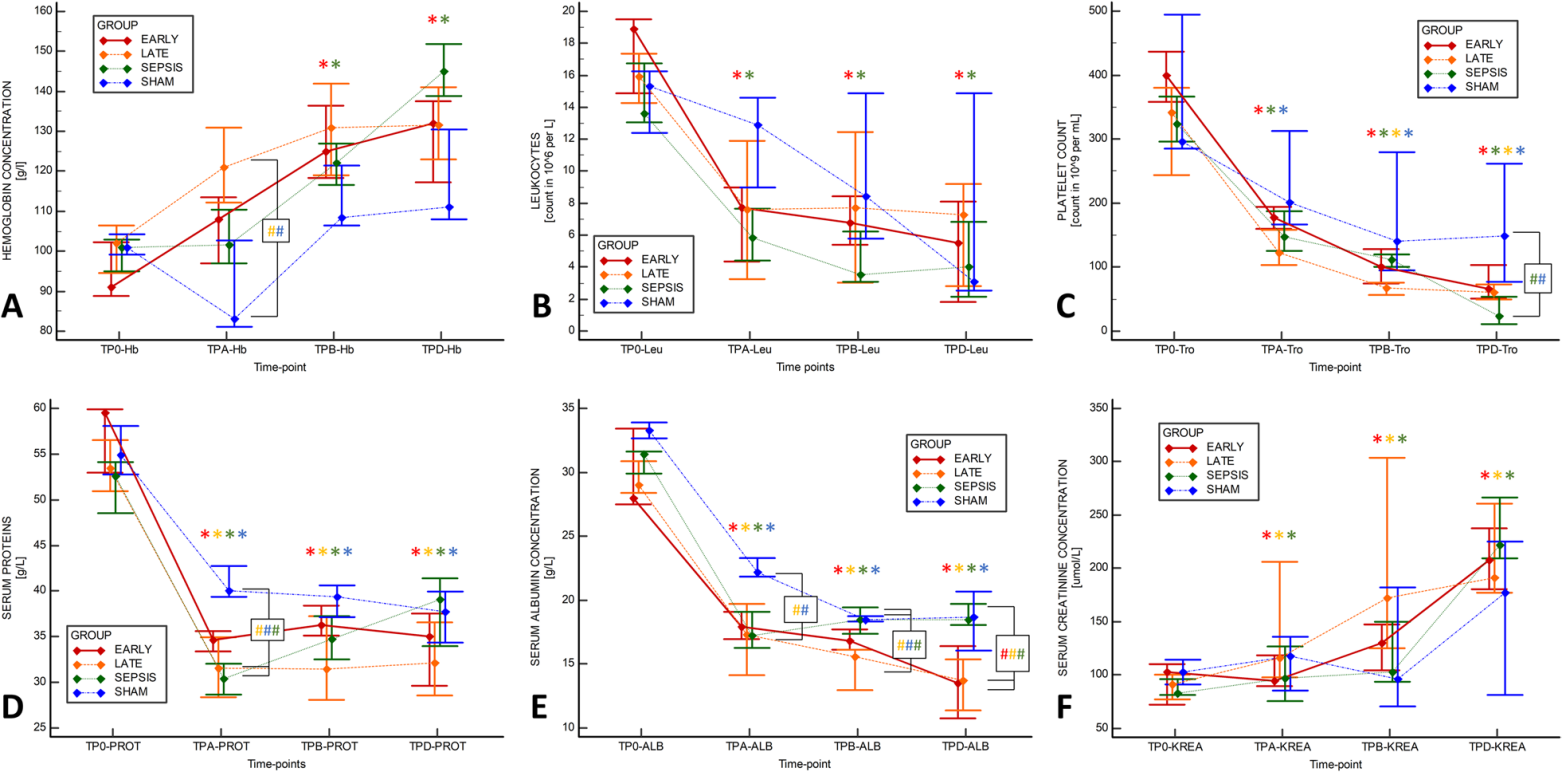
Evolution of hematologic and laboratory parameters. **A** Hemoglobin [g/dL], **B** Leukocytes [count in 10^6^ in L], **C** Platelets [count in 10^9^ per L], **D** Plasma protein level [g/L], **E** Albumin level [g/L], **F** Plasma creatinine level [μmol/L]. The graph displays the median (diamond) and 25–75% quartile range with connecting lines. *TP-0* start of the experiment/fecal inoculation, *TP-A* start of the HA or defined equivalent, *TP-B* 6 h of the HA treatment, *TP-D* last measureable value. Asterisk (*) in adequate color marks significance vs. baseline (RM ANOVA: Friedman test); Hashtag (#) in corresponding colors marks significant intergroup difference in given time-point (Kruskal Wallis ANOVA on ranks)

After fecal inoculation, progressive sepsis with vasoplegic shock evolved in all animals in the septic groups (Fig. 2, Additional file 1: Table S1). Fluid resuscitation and norepinephrine were needed to maintain the volume status and MAP ≥ 65 mmHg. The time to HA initiation did not differ between the EARLY and LATE animals (1260 (1080–1395) vs. 1200 (1035–1410) min, *p* = 0.8534). Our analyses revealed no positive effect of HA on either the requirement for vasopressor medication or the progressive hemodynamic deterioration (primary analysis: Fig. 2, Additional file 1: Table S1; pairwise comparison between SEP-HA (joint EARLY and LATE) vs. SEPSIS group: Additional file 1: Table S2). After the HA initiation, the EARLY animals reached norepinephrine infusion rates of 0.3 and 0.5 μg/kg/min significantly faster than that in other septic animals that were not treated with HA at that point (SEPSIS and LATE) (Table 1, Fig. 3). The rate of escalation after reaching a norepinephrine infusion rate of 0.5 μg/kg/min was similar between all septic groups. The resuscitative fluids volume and overall fluid balance did not differ between groups (Table 1). Despite this rather liberal fluid substitution, markers of inadequate circulating blood volume (decreased stroke volume, low GEDV, high PPV) were observed in the HA-treated septic animals. Rise in extravascular lung water and pulmonary vascular permeability indexes speak for increased permeability and development of interstitial tissue edema potentially facilitated by a significant drop in albumin concentration (Fig. 4, Additional file 1: Tables S1 and S2).

### Effects on inflammatory and biochemical markers of HA in sepsis

**Fig. 5:**
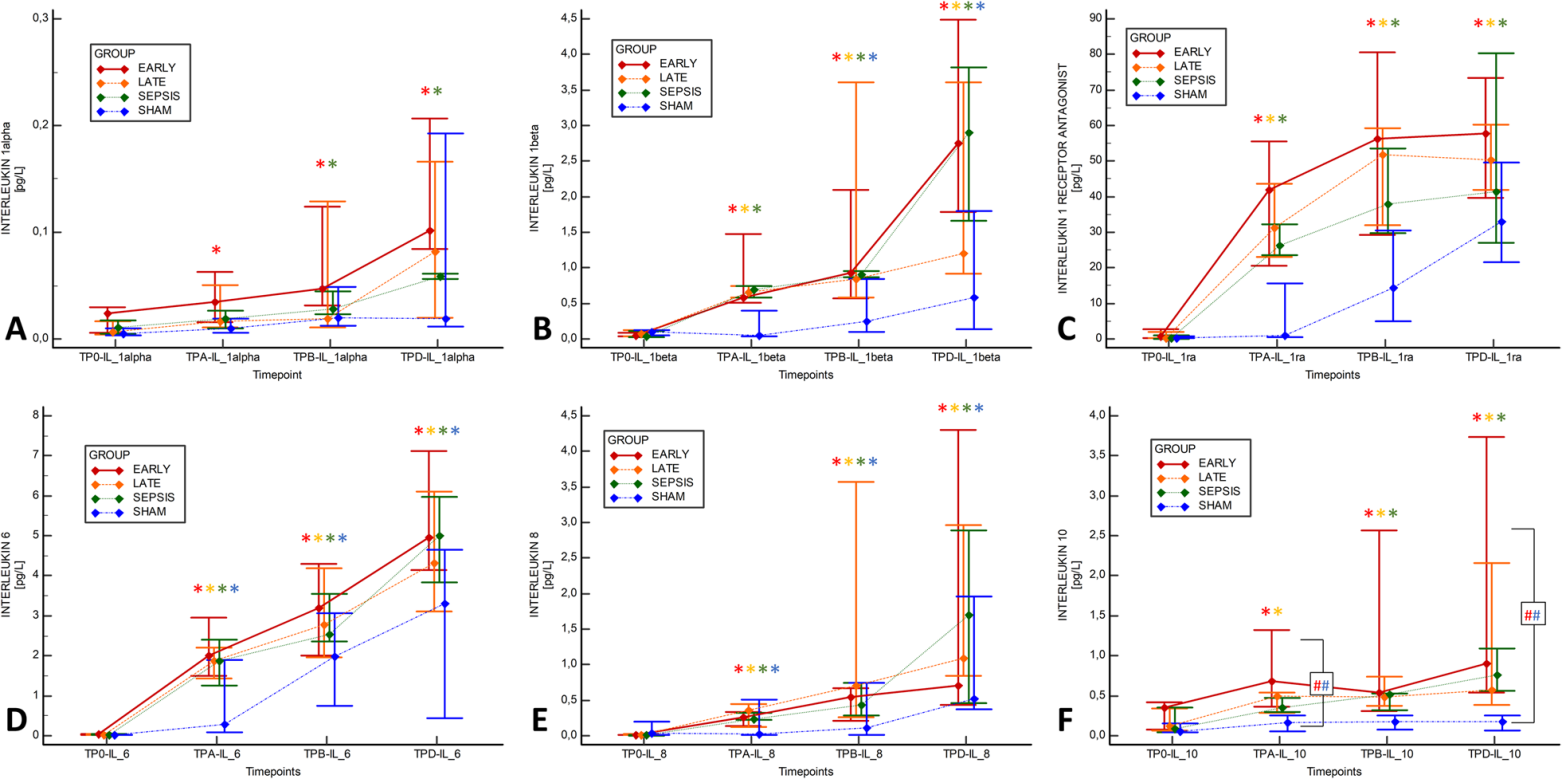
Evolution of cytokine levels. **A** Iinterleukin-1α, **B** Interleukin-1β, **C** Interleukin-1 receptor antagonist (IL-1RA), **D** Interleukin-6, **E** Interleukin-8, **F** Interleukin-10. The graph displays the median (diamond) and 25–75% quartile range with connecting lines. *TP-0* start of the experiment/fecal inoculation, *TP-A* start of the HA or defined equivalent, *TP-B* 6 h of the HA treatment, *TP-D* last measureable value. Asterisk (*) in adequate color marks significance vs. baseline (RM ANOVA: Friedman test); Hashtag (#) in corresponding colors marks significant intergroup difference in given time-point (Kruskal Wallis ANOVA on ranks)

Inflammatory mediators increased throughout the experiment in all animals in the septic groups without any intergroup difference (Fig. 5, Additional file 1: Table S1) or impact of the HA. The leukocyte and platelet drop and rise in hemoglobin were similar among the animals in the septic groups (Fig. 4, Additional file 1: Table S1). Albumin and protein concentration decreased progressively in all the groups, but the decrease was more significant in the HA-treated animals, especially during the late course. Renal function markers (blood urea nitrogen, creatinine) increased in all animals in the septic groups, and all the animals (including SHAM) fulfilled the criteria for the first and mostly for the second level of acute kidney injury. With the rise in lactate concentration, metabolic acidosis occurred in all septic animals without any intergroup difference. In general, no evidence of positive HA impact on laboratory markers among the animals in the septic groups has been identified (Additional file 1: Tables S1 and S2).

### HA effects in sham-operated healthy animals

After the HA initiation, all SHAM animals developed hyperdynamic vasoplegia as evidenced by tachycardia, elevated CO, and reduced SVR (Fig. 2, Additional file 1: Table S1 and S3), and needed vasopressor support to maintain MAP (Fig. 3). However, only minor derangement in acidobasis (pH, base excess, lactate) was observed. The exposure of healthy animals to HA resulted in a gradual reduction in circulating leukocytes, platelets, and hemoconcentration, and decreasing levels of total protein and albumin (Additional file 1: Tables S1 and S3). These changes were not observed among sham-operated historical CONTROL animals without HA (Additional file 1: Table S3). Finally, gradually increased plasma levels of various cytokines unopposed by the HA were observed (Fig. 5, Additional file 1: Table S3).

## Discussion

Our large-animal randomized controlled experiment investigating the effects of Cytosorb HA in fecal peritonitis-induced sepsis did not demonstrate protection of animals from deterioration in fulminant sepsis, regardless of whether HA was initiated in the early or advanced phase of septic shock. In contrast, the HA resulted in a more aggressive vasopressor load and worse survival. Finally, HA in healthy animals resulted in hemodynamic deterioration, systemic inflammatory response, and cytopenia.

For over a decade, the critical care community has been discussing the immune response modulation and mediators adsorbing devices used in sepsis. Currently, six devices are marketed with different adsorption capabilities and research backgrounds [10]. The CytoSorb device stands out due to its demonstrably strong in vitro efficacy [19, 20], the number of clinical runs, and the evidence gathered over this time. CytoSorb decreased the levels of inflammatory markers, improved the hemodynamic parameters, and prolonged survival in small animals after cecal ligation puncture [21, 22] and fatal endotoxemia [23]. Based on such preliminary experimental data an over-optimistic clinical application has started. However, experiments on rodents cannot be directly clinically replicable on humans and the current evidence from human-based studies remains extremely inconsistent. Koehler et al. recently reviewed the evidence from 170 studies, analyzing the indications, possible effects, and research findings [24]. Besides the potential for eliminating exotoxins (anticoagulants) or endogenous substances (bilirubin, myoglobin, or free hemoglobin), the vasoplegic shock owing to systemic inflammation holds the greatest clinically relevance. Such effect has been observed in several observational studies and a recent meta-analysis of 33 studies by Hawchar et al. [3] reported a positive impact on norepinephrine dose and hemodynamic stability. An overall Hedge’s g index of 1.64 should indicate a large clinical effect, but the population comprised 140 patients from four controlled trials, of which only one was randomized. Unlike this one randomized trial with 20 septic shock patients [6], in two much larger trials, no significant improvement by the HA was observed [5, 7]. Schadler et al. [5] randomized 97 patients with septic shock, but was unable to demonstrate any effect on systemic levels of interleukin-6; unmatched mortality was higher in the treatment group (effect, which disappeared after adjustment). Another randomized multicenter pilot compared 23 CS-treated patients with 26 controls [7]. The primary outcome of this study was negative (vasoplegic shock resolution). Besides, it was negative in multiple secondary endpoints including systemic levels of inflammatory markers, catecholamine requirements, and adverse events rate. During the COVID-19 pandemic, several small randomized controlled trials were used to test the application of CS to decrease the impact of inflammation without any meaningful effect [7, 8, 25]. This conflicting evidence may be a result of our poor understanding of the actual mechanisms and the role of inflammatory mediators during septic shock, and a lack of rational treatment dose and timing.

Based on the in vitro data, CS allows the adsorption of significant amounts of substances ranging from 5 to 55 kDa. The removal rate for most interleukins was above 90% after 120 min exposition [19]. However, in vivo, such an effect was not always observed. In a pig model of burn injury, CS led to a decrease in the inflammatory markers in the circuit without affecting the systemic concentrations [26]. Because the concentrations of the pre-post filter mediators differ, the dose delivered may be inadequate to cause any significant effect. Schultz et al. demonstrated that a higher delivery dose was favorable, while a low dose was not [27]. Based on the Schultz equation [27] the delivery dose in the Linden study [26] was 2.25 L/kg (one 300 mL CS cartridge per 6-h treatment with a 250 mL/min blood flow in a 40 kg pig). In our study, the delivery dose was 2.4 L/kg (one 300 mL CS cartridge per 10-h treatment with a blood flow rate of 200 mL/min in a 50 kg pig). However, in the study by Schultz [27], the higher delivery dose was based on prolonged and multiple device runs rather than high flow rates. Therefore, the initial effect of CS should consider the blood flow and body weight only, and it was 375 mL/kg during the first hour in a study by Linden et al. [26] and 240 mL/kg in our experiment. This means that the doses are two-to-three times more intensive as compared to normal human treatments with 150 mL/h blood flows and a weight of 80 kg (112.5 mL/kg). One may hypothesize that the mediator production in unopposed peritoneal sepsis may exceed the device capacity. This seems unlikely because the systemic concentrations between the treated groups and septic controls did not differ significantly both in our study and the study by Linden et al. [26]. Besides Linden [26] demonstrated the pre-post filter decrease even after 6 h of the CS run.

The impact of the HA timing was one of the primary aims of our trial. In several studies, early-initiated HA was associated with better results [28–30]. On the contrary, in current praxis, HA is considered the last resort in irreversible vasoplegia. A dose of norepinephrine above 0.5 μg/kg/min in patients with adequate fluid loading and cardiac output was considered as a trigger by Hawchar et al. [6]. A recent meta-analysis reported a similar pre-treatment norepinephrine dose (median 0.55 (0.39–0.9) μg/kg/min) [3]. In our EARLY animals, the HA led to immediate norepinephrine rate escalation, and the survival time after HA initiation was significantly shorter in the LATE animals, despite similar sepsis initiation time. Moreover, the survival time of the septic animals without HA was the longest. From this point of view, our data do not support “the earlier, the better” notion and even raise certain safety concerns. In our experiment, HA seems to propagate vasoplegia, increase endothelial permeability, and negatively affect the platelets and leukocyte counts and circulating protein levels. No convincing explanation exists for our observation. The ability of blood-biomaterial interaction to activate either the humoral and cellular host response (i.e., bio-incompatibility) or remove beneficial substances should be taken into consideration. The increase in inflammatory mediators and drop in albumin levels observed in our animals supports the plausibility of these mechanisms. However, we did not specifically address this issue by pre- and post-cartridge samples. On the contrary, the safety signal observed in our study is in line with the results of two prospective studies demonstrating potential HA-induced harm [31].

### Limitations

Our experiment has several limitations, which may have affected our results and influenced extrapolation to humans. First, the fact that human and pig native immunology reactivity to insults may differ has to be taken into account. Besides, animal aging does not directly correspond to human development, and hence it may significantly differ from matured or senescent human pathophysiology. Further, it was an open-label study, but the strict experimental protocol should have reduced any sources of experimental bias, and blinding was imposed on data analysts (all biochemical data analyses).

No source control and antimicrobials were provided. Our model was designed to create hyperdynamic sepsis, with a progressive increase in severity over time. Antibiotic therapy was expected to blunt the host response, thereby attenuating the development and full manifestation of a true clinical septic response over 24 h, which is what we instead sought to elicit in our experiment. Such ongoing inflammatory stimulus may overcome the HA's ability to counteract. However, such a situation may not be that rare in clinical routine. Source control is not always possible as demonstrated previously [32] and administration of inadequate antimicrobials may still occur. Prior research suggests that HA can affect antimicrobial levels [33], introducing further unwanted uncertainties into our experiment.

To reduce the number of experimental animals we did not include a sham-operated HA untreated CONTROL group. To analyze the unwanted (and previously not expected) HA effects in the SHAM animals, we used a historical control from a previous experiment [18]. Since our methodology has remained consistent over a large period, we believe it does not introduce errors in our results.

Finally, a potential limitation of our study was the small number of experimental animal subjects. Such a complex experimental approach is expensive, highly time-demanding, and requires adequately trained critical care staff; hence, precludes using high numbers of animals. This limits the use of robust statistical methods. However, it enables us to study in detail the underlying pathophysiology on individual subject level and create extreme conditions (i.e., no-source-control fulminant sepsis); a process never possible in human subjects, but potentially useful to prove or refute certain hypothetical treatment effects. For instance, the time to initiate HA therapy in the LATE group was shorter than that in the EARLY group, which may indicate divergent sepsis dynamics on the individual level; however, the HA effect was not diverse. We believe that our experimental approach still provides a valuable alternative to fill the actual knowledge gap in settings where adequately large randomized clinical trials are difficult to conduct. Encouragingly, all pigs that underwent Cytosorb treatment demonstrated consistent non-beneficial responses. Thus, it is unlikely that increasing the sample size would have altered the results.

## Conclusions

In this large-animal-controlled fulminant sepsis study, the CytoSorb HA was not able to counteract the progression of the disease in the early or more advanced phase of septic shock. The systemic levels of inflammatory markers were unaffected by any of the treatment timings. On the contrary, findings from the sham-operated non-septic HA-exposed animals suggest potential safety concerns.

### Supplementary Information

**Additional file 1: Table S1.** Hemodynamic, hematologic, laboratory parameters and cytokine levels (overall statistics). **Table S2. **Hemodynamic, hematologic, laboratory parameters and cytokine levels (septic animals exposed to HA versus septic animals without HA). **Table S3. **Hemodynamic, hematologic, laboratory parameters and cytokine levels (sham operated animals exposed to HA versus historic controls).

## Data Availability

The processed data are available as supplemental material to this article (*link*). The raw data are available at the corresponding author upon reasonable request.

## Abbreviations

CO: Cardiac output
CONTROL: Historical control group without sepsis and adsorption treatment
CS: CytoSorb
EARLY: Study group with sepsis and early adsorption treatment
EVLW: Extravascular lung water
GEDV: Global end-diastolic volume
HA: Hemoadsorption
LATE: Study group with sepsis and late adsorption treatment
MAP: Mean arterial pressure
NE: Norepinephrine/noradrenaline
PPV: Pulse pressure variation
PVPI: Pulmonary vasculature permeability index
SEP-HA: Combined group of septic animals with adsorption treatment
SEPSIS: Study group with sepsis but without adsorption treatment
SHAM: Control group with adsorption treatment
SVR: Systemic vascular resistance
TP-0: Timepoint zero: baseline measurement
TP-A: Timepoint at the initiation of HA treatment or equivalent
TP-B: Six hours after HA initiation or equivalent
TP-D: The last measureable set of values before death/end of experiment
