# Supplementary material for: Ineffectiveness of hemoadsorption in large animals with abdominal sepsis: a randomized controlled porcine study
Source: Intensive Care Med Exp. 2024 Apr 18;12:38. doi: 10.1186/s40635-024-00622-x (PMC11026308; doi:10.1186/s40635-024-00622-x)
Supplement: Supplementary file 1 — Additional file 1: Table S1. Hemodynamic, hematologic, laboratory parameters and cytokine levels (overall statistics). Table S2. Hemodynamic, hematologic, laboratory parameters and cytokine levels (septic animals exposed to HA versus septic animals without HA). Table S3. Hemodynamic, hematologic, laboratory parameters and cytokine levels (sham operated animals exposed to HA versus historic controls). [file 40635_2024_622_MOESM1_ESM.docx]

**Supplemental digital content:**

1. ARRIVE checklist
2. Table S1 – Hemodynamic, hematologic, laboratory parameters and cytokine levels (overall statistics)
3. Table S2 – Hemodynamic, hematologic, laboratory parameters and cytokine levels (septic animals exposed to HA versus septic animals without HA)
4. Table S3 – Hemodynamic, hematologic, laboratory parameters and cytokine levels (sham operated animals exposed to HA versus historic controls)

| **Table S1 – Hemodynamic, hematologic, laboratory parameters and cytokine levels (overall statistics)** | | | | | | | | |
| --- | --- | --- | --- | --- | --- | --- | --- | --- |
|  | **TP -0** | | **TP-A** | | **TP-B** | | **TP-D** | |
|  | **Median** | **25 - 75**  **percentile** | **Median** | **25 - 75 percentile** | **Median** | **25 - 75**  **percentile** | **Median** | **25 - 75**  **percentile** |
| **HEART RATE** (beats/minute) | | | | | | | | |
| EARLY | **100** | **91 - 119** | **143 BL** | **91 – 160** | **196 BL, A** | **173 - 219** | **190 BL,A** | **174 - 202** |
| LATE | **88** | **72 - 96** | **130 BL** | **109 - 137** | **185 BL,A** | **147 - 225** | **197 BL,A** | **138 - 217** |
| SEPSIS | **93** | **84 - 109** | **119** | **82 - 142** | **210 BL,A** | **156 – 219** | **268 *,BL,A** | **244 - 283** |
| SHAM | **103** | **99 - 112** | **63** | **61 - 100** | **116 A** | **109 - 186** | **179 *,BL,A** | **128 - 205** |
| **MEAN ARTERIAL PRESSURE** (mmHg) | | | | | | | | |
| EARLY | **86** | **75 - 94** | **63 BL** | **63 - 64** | **71** | **66 - 72** | **50 BL,B** | **41 - 65** |
| LATE | **88** | **72 - 91** | **65 BL** | **65 - 69** | **58 #,BL** | **52 - 65** | **59 BL,A** | **46 - 63** |
| SEPSIS | **74** | **66 - 87** | **63 BL** | **60 - 66** | **71 #,A** | **65 - 71** | **58 BL,A,B** | **51 - 60** |
| SHAM | 81 | 72 - 87 | 64 | 61 - 66 | 65 | 64 - 66 | 62 | 54 - 66 |
| **MEAN PULMONARY ARTERIAL PRESSURE** (mmHg) | | | | | | | | |
| EARLY | 19 | 18 - 22 | 26 | 23 - 29 | 25 | 18 - 30 | 36 | 20 - 40 |
| LATE | **17** | **16 - 20** | **25 BL** | **23 - 28** | **34 $.BL.A** | **29 - 40** | **34 BL.A** | **30 - 40** |
| SEPSIS | **18** | **16 - 20** | **28 BL** | **21 - 29** | **22 $** | **18 - 24** | **36 BL** | **25 - 43** |
| SHAM | **19** | **17 - 21** | **23 BL** | **20 - 32** | **33 BL** | **29 - 39** | **36 BL.A.B** | **26 - 38** |
| **CENTRAL VENOUS PRESSURE** (mmHg) | | | | | | | | |
| EARLY | **10** | **8 - 14** | **16 BL** | **16 - 18** | **17 BL** | **14 - 19** | **16 BL** | **13 - 19** |
| LATE | **10** | **9 - 11** | **17 BL** | **15 - 18** | **14 BL.A** | **12 - 16** | **15 BL.A** | **13 - 16** |
| SEPSIS | **10** | **9 - 14** | **20 BL** | **15 - 21** | **18 BL** | **15 - 20** | **19 BL** | **18 - 21** |
| SHAM | **10** | **9 - 12** | **15 BL** | **15 - 18** | **16 BL.A** | **15 - 18** | **21 BL.A.B** | **17 - 22** |
| **PULMONARY ARTERIAL OCCLUDED PRESSURE** (mmHg) | | | | | | | | |
| EARLY | **9** | **8 - 10** | **12 BL** | **11 - 13** | **11 BL.A** | **9 - 12** | **17 BL.A.B** | **13 - 18** |
| LATE | **8** | **7 - 9** | **11 BL** | **9 - 13** | **11 BL.A** | **10 - 12** | **11 BL.A** | **10 - 12** |
| SEPSIS | **8** | **7 - 9** | **12 BL** | **12 - 14** | **13 BL** | **11 - 13** | **16 BL.A.B** | **13 - 18** |
| SHAM | **8** | **7 - 9** | **13 BL** | **11 - 14** | **11 BL** | **9 - 13** | **10 BL** | **8 - 14** |
| **CARDIAC OUTPUT** (l/min) | | | | | | | | |
| **EARLY** | **3.2** | **3.0 - 3.5** | **4.7 BL** | **3.0 - 5.3** | **4.2 BL** | **3.6 - 4.7** | **1.8 BL.A.B** | **1.8 - 2.9** |
| LATE | 3.1 | 3.0 - 3.5 | 4.0 | 3.7 - 5.6 | **3.2 $** | **1.3 - 4.4** | 2.1 | 1.7 - 2.7 |
| SEPSIS | **3.5** | **2.8 - 3.6** | 4.8 | 3.4 - 6.0 | **7.1 $.BL** | **5.5 - 8.8** | **6.1 BL** | **3.6 - 6.7** |
| SHAM | 3.2 | 3.0 - 3.7 | 2.6 | 2.6 - 3.7 | 4.9 | 3.5 - 5.2 | 3.9 | 3.3 - 5.1 |
| **WEIGHTED CARDIAC OUTPUT** (ml/min/kg) | | | | | | | | |
| EARLY | **64** | **59 - 68** | **93 BL** | **57 - 108** | **84 BL** | **69 - 95** | **42 BL.A.B** | **34 - 55** |
| LATE | 69 | 62 - 72 | 95 | 75 - 114 | 74 | 27 - 86 | 38 | 33 - 57 |
| SEPSIS | **59** | **56 - 68** | 95 | 72 - 103 | **134 BL** | **107 - 168** | **109 BL** | **75 - 114** |
| SHAM | 63 | 56 - 75 | 51 | 45 - 77 | 86 | 75 - 89 | 77 | 56 - 100 |
| **STROKE VOLUME** (ml) | | | | | | | | |
| EARLY | **34** | **29 - 36** | **33 BL** | **32 - 37** | **21 BL** | **20 - 24** | **10 BL.A.B** | **9 - 11** |
| LATE | 38 | 32 - 52 | 34 | 29 - 51 | **15 $** | **7 - 22** | 12 | 7 - 16 |
| SEPSIS | 38 | 29- 40 | 40 | 38 - 45 | **35 $.BL** | **34 - 40** | **23 BL** | **14 - 28** |
| SHAM | 31 | 28 - 35 | 43 | 39 - 44 | 31 | 28 - 36 | 26 | 16 - 39 |
| **SYSTEMIC VASCULAR RESISTANCE** (dyn·s/cm^5^) | | | | | | | | |
| EARLY | **1966** | **1499 - 1975** | **824 BL** | **677 - 1281** | **971 BL** | **887 - 1200** | **1188 BL** | **1156 - 1308** |
| LATE | 1727 | 1564 - 2200 | 894 | 688 - 1133 | 1073 | 961 - 2026 | 1368 | 1145 - 1588 |
| SEPSIS | **1723** | **1205 - 2062** | **730** | 678 - 990 | **586 BL** | **411 - 827** | **525 BL** | **514 - 879** |
| SHAM | **1929** | **1353 - 1995** | 1343 | 1105 - 1584 | **769 BL** | **713 - 1187** | **800 BL** | **646 - 1352** |
| **PULMONARY VASCULAR RESISTANCE** (dyn·s/cm^5^) | | | | | | | | |
| EARLY | **471** | **410 - 540** | **272** | **143 - 423** | **303 BL** | **200 - 376** | **600 A.B** | **313 - 970** |
| LATE | 400 | 380 - 478 | 240 | 202 - 286 | **969 $** | **558 - 1930** | 1050 | 675 - 1291 |
| SEPSIS | 400 | 343 - 482 | 212 | 153 - 319 | **90 $** | **56 - 196** | 246 | 151 - 341 |
| SHAM | 413 | 366 - 535 | 277 | 254 - 395 | 392 | 320 - 433 | 369 | 304 - 647 |
| **GLOBAL END-DIASTOLIC VOLUME INDEX** (ml/kg) | | | | | | | | |
| EARLY | **10.3** | **9.9 - 11.0** | 9.3 | 8.3 - 10.0 | **6.2 BL** | **5.8 – 8.0** | 5.8 | 5.2 - 6.4 |
| LATE | 10.5 | 9.8 - 13.5 | 9.6 | 8.7 - 10.9 | 9.7 | 9.1 - 10.0 | N/A | N/A |
| SEPSIS | 10.0 | 9.6 - 10.2 | 10.5 | 9.3 – 11.4 | 9.4 | 8.9 - 10.5 | 9.0 | 8.4 - 9.5 |
| SHAM | 11.6 | 8.1 - 11.8 | 9.0 | 8.6 - 12.7 | 9.9 | 8.7 - 10.8 | 11.3 | 9.3 - 11.6 |
| **EXTRA-VASCULAR LUNG WATER INDEX** (ml/kg) | | | | | | | | |
| EARLY | 7.9 | 7.4 - 8.5 | 8.6 | 8.1 - 9.2 | 7.5 | 6.6 - 7.8 | 19.6 | 10.2 - 28.9 |
| LATE | 7.9 | 7.0 - 9.6 | 8.2 | 7.6 – 11.0 | 17.7 | 7.4 – 28.0 | N/A | N/A |
| SEPSIS | 7.4 | 7.1 - 8.1 | 8.9 | 8.5 - 9.5 | 8.4 | 7.3 - 9.4 | 8.5 | 7.5 - 9.4 |
| SHAM | 7.8 | 7.3 - 9.2 | 10.3 | 8.8 - 10.7 | 9.2 | 7.9 - 10.7 | 9.5 | 9.5 - 10.2 |
| **PULMONARY VASCULATURE PERMEABILITY INDEX** | | | | | | | | |
| EARLY | **3.2** | **2.9 - 3.3** | **3.7 BL** | **3.5 - 3.9** | **3.8 BL** | **3.4 - 5.2** | 7.9 | 7.9 - 7.9 |
| LATE | 3.0 | 2.4 - 3.2 | 3.5 | 3.0 - 4.1 | 6.1 | 3.3 - 8.8 | N/A | N/A |
| SEPSIS | 3.1 | 2.9 - 3.5 | 3.5 | 3.2 - 3.7 | 3.2 | 3.1 - 4.1 | 3.8 | 3.5 – 4.0 |
| SHAM | 3.2 | 3.2 - 4.6 | 3.7 | 3.1 - 4.4 | 4.3 | 3.5 - 4.6 | 3.4 | 3.3 - 4.5 |
| **LEFT VENTRICULAR CONTRACTILITY (dPmax)** (mmHg/s) | | | | | | | | |
| EARLY | **696** | **617 - 865** | **765 BL** | **637 - 881** | **2371 BL,A** | **1800 - 2727** | **2678 BL,A,B** | **1994 - 3206** |
| LATE | 753 | 587 - 799 | 928 | 661 - 1326 | 1861 | 1497 - 2225 | 2707 | 2542 - 2872 |
| SEPSIS | 860 | 496 - 913 | **725** | **570 - 755** | **1360 A** | **1235 - 1647** | **1922 A** | **1144 - 2700** |
| SHAM | **628** | **546 - 692** | **695** | **487 - 739** | **1337 BL,A** | **1037 - 1849** | **1913 BL,A** | **1368 - 1970** |
| **CARDIAC FUNCTION INDEX** (unit per minute) | | | | | | | | |
| EARLY | 7.6 | 6.5 - 7.9 | 12.0 | 7.1 – 12.0 | 13.0 | 8.0 – 15.0 | 7.6 | 6.9 - 8.2 |
| LATE | 6.1 | 4.7 - 7.1 | 10.3 | 8.2 - 12.5 | 6.0 | 0.0 – 12.0 | N/A | N/A |
| SEPSIS | **6.9** | **6.2 - 7.7** | **11.0** | **6.8 – 12.0** | **11.6 BL,A** | **9.2 – 14.0** | 14.5 | 14.0 – 15.0 |
| SHAM | **6.6** | **5.0 - 7.3** | **5.7** | **5.3 - 9.6** | **11.0 BL,A** | **9.0 - 11.5** | **12.0 BL,A,B** | **9.2 - 13.5** |
| **PULSE PRESSURE VARIATION** (%) | | | | | | | | |
| EARLY | **12** | **8 - 15** | **20** | **11 - 22** | **29 *,BL,A** | **28 - 31** | **20 BL,B** | **12 - 25** |
| LATE | 12 | 10 - 13 | 17 | 15 - 18 | 17 | 17 - 17 | 21 | 10 - 32 |
| SEPSIS | **12** | **8 - 13** | **12** | **10 - 21** | **17 BL,A** | **13 - 22** | **26 BL** | **20 - 32** |
| SHAM | 12 | 10 - 15 | 14 | 7 - 17 | **12 *** | **11 - 14** | 10 | 9 - 30 |
| **SERUM LACTATE LEVEL** (mmol/L) | | | | | | | | |
| EARLY | **1.0** | **1.1 - 1.5** | **1.3** | **1.1 - 1.4** | **3.9 *,BL,A** | **2.7 - 4.6** | **13.3 *,BL,A,B** | **10.3 - 15.9** |
| LATE | **1.1** | **1.0 - 1.2** | **1.5** | **1.2 - 1.6** | **3.0 BL,A** | **2.1 - 3.9** | **10.9 BL,A,B** | **6.2 - 16.6** |
| SEPSIS | **1.3** | **1.0 - 1.7** | **1.1** | **1.0 - 1.2** | **2.1 A** | **1.8 - 2.9** | **8.6 BL,A,B** | **6.6 - 11.6** |
| SHAM | 1.1 | 1.0 - 1.2 | 1.1 | 0.8 - 1.7 | **1.7 *** | **1.2 - 1.8** | **3.4 *** | **1.7 - 5.9** |
| **MIXED VENOUS OXYGEN SATURATION (S_V_O_2_)** (%) | | | | | | | | |
| EARLY | 56.4 | 49.4 - 62.6 | 56.0 | 41.9 - 63.7 | 58.7 | 51.8 - 66.9 | 35.3 | 29.0 - 43.5 |
| LATE | 57.5 | 51.8 - 60.5 | 58.0 | 53.1 - 62.5 | 62.0 | 37.0 - 63.5 | 36.0 | 32.8 - 45.5 |
| SEPSIS | 54.6 | 46.5 - 63.0 | 57.0 | 28.0 - 60.6 | 58.9 | 55.2 - 61.6 | 33.3 | 30.0 - 36.6 |
| SHAM | **54.4** | **52.8 - 55.6** | **46.8 BL** | **39.3 - 52.6** | **63.5 A** | **45.7 - 68.5** | **60.0 A** | **42.4 - 64.4** |
| **HOROWITZ INDEX (PaO_2_/FiO_2_)** (mmHg) | | | | | | | | |
| EARLY | **473** | **440 - 512** | **428** | **376 – 451** | **334 BL,A** | **296 - 405** | **355 BL,A** | **332 - 410** |
| LATE | 475 | 411 - 508 | 360 | 339 - 449 | 454 | 357 – 457 | 364 | 293 – 408 |
| SEPSIS | **470** | **456 - 477** | **430 BL** | **376 - 457** | **370 BL** | **344 - 404** | **385 BL** | **356 – 434** |
| SHAM | **479** | **474 - 507** | 445 | 243 - 486 | **352 BL** | **202 - 470** | **225 BL** | **193 - 461** |
| **ARTERIAL pH** | | | | | | | | |
| EARLY | **7.52** | **7.50 - 7.55** | **7.41 BL** | **7.38 - 7.42** | **7.29 BL,A** | **7.14 - 7.31** | **6.94 *,BL,A,B** | **6.88 - 6.99** |
| LATE | **7.53** | **7.51 - 7.58** | **7.39 BL** | **7.38 - 7.41** | **7.29 BL,A** | **7.27 - 7.29** | **7.00 *,BL,A,B** | **6.88 - 7.09** |
| SEPSIS | **7.54** | **7.51 - 7.56** | **7.42 BL** | **7.40 - 7.46** | **7.34 BL,A** | **7.26 - 7.38** | **7.03 BL,A,B** | **6.98 - 7.09** |
| SHAM | **7.53** | **7.51 - 7.54** | **7.46** | **7.38 - 7.49** | **7.39 BL,A** | **7.34 - 7.44** | **7.30 *,BL,A,B** | **7.21 - 7.36** |
| **ARTERIAL BASE EXCESS** (mmol/L) | | | | | | | | |
| EARLY | **6.5** | **4.4 - 6.8** | **0.7 BL** | **-0.4 - 1.8** | **-5.7 BL,A** | **-10.8 - -3.7** | **-22.8 *.BL,A,B** | **-24.8 - -21.0** |
| LATE | **7.8** | **5.6 - 9.1** | **-0.5 BL** | **-2.0 - 0.8** | **-5.9 BL,A** | **-7.1 - -5.0** | **-20.7 *,BL,A,B** | **-24.8 - -13.7** |
| SEPSIS | **6.2** | **4.2 - 8.2** | **1.8** | **1.1 - 2.5** | **-1.0 BL,A** | **-2.5 - -0.7** | **-16.9 BL,A,B** | **-18.5 - -13.2** |
| SHAM | **5.3** | **4.5 - 7.2** | **2.1 BL** | **0.4 - 2.6** | **0.2 BL,A** | **-2.4 - 0.9** | **-2.9 * BL,A,B** | **-8.1 - 0.3** |
| **HEMOGLOBIN CONCENTRATION** (g/dL) | | | | | | | | |
| EARLY | **91** | **89 - 102** | **108 BL** | **97 – 114** | **125 BL,A** | **118 – 137** | **132 BL,A** | **117 – 138** |
| LATE | 102 | 95 - 107 | **121 *** | **112 – 131** | 131 | 119 – 142 | 132 | 123 – 141 |
| SEPSIS | **101** | **95 - 103** | **102** | **97 – 111** | **122 BL,A** | **117 - 127** | **145 BL,A,B** | **139 - 152** |
| SHAM | 101 | 99 - 104 | **83 *** | **81 - 103** | **109 A** | **107 - 122** | **111 A** | **108 - 131** |
| **LEUKOCYTE COUNT** (10^9^/L) | | | | | | | | |
| EARLY | **18.9** | **14.9 - 19.5** | **7.7 BL** | **4.4 – 9.0** | **6.8 BL** | **5.4 - 8.5** | **5.5 BL** | **1.8 - 8.1** |
| LATE | 16.0 | 14.0 - 17.4 | 7.6 | 3.0 - 11.9 | 7.7 | 3.0 - 12.5 | 7.3 | 2.8 - 9.2 |
| SEPSIS | **13.6** | **13.1 - 16.7** | **5.9 BL** | **4.4 - 7.7** | **3.5 BL** | **3.1 - 6.2** | **4.0 BL,A** | **2.2 - 6.8** |
| SHAM | 15.3 | 12.4 - 16.3 | 12.9 | 9.0 - 14.6 | 8.5 | 5.8 - 14.9 | 3.1 | 2.6 - 14.9 |
| **PLATELET COUNT** (10^9^/L) | | | | | | | | |
| EARLY | **399** | **359 - 437** | **178 BL** | **160 – 194** | **101 BL,A** | **74 - 128** | **66 BL,A,B** | **51 - 103** |
| LATE | **342** | **244 – 381** | 123 | 104 – 159 | **68 BL** | **57 – 76** | **61 BL** | **50 - 73** |
| SEPSIS | **324** | **297 - 367** | **148 BL** | **125 – 187** | **112 BL,A** | **101 – 121** | **24 *,BL,A,B** | **11 - 54** |
| SHAM | **296** | **285 - 495** | **201 BL** | **167 - 313** | **141 BL,A** | **95 - 280** | **149 *,BL,A,B** | **77 - 262** |
| **SERUM PROTEIN CONCENTRATION** (g/L) | | | | | | | | |
| EARLY | **59.5** | **52.9 - 59.9** | **34.6 BL** | **33.4 - 35.6** | **36.3 BL** | **35.1 - 38.4** | **35.0 BL** | **29.7 - 37.5** |
| LATE | 53.5 | 51.0 - 56.5 | **31.6 *** | **28.4 - 34.9** | **31.5 BL** | **28.1 - 37.2** | **32.1 BL** | **28.6 - 36.6** |
| SEPSIS | **52.6** | **48.6 - 54.1** | **30.4 *,BL** | **28.7 – 32.0** | **34.7 BL,A** | **32.5 - 37.2** | **39.1 BL,A** | **34.0 - 41.4** |
| SHAM | **54.9** | **52.8 - 58.1** | **40.0 *,BL** | **39.3 - 42.7** | **39.4 BL,A** | **37.2 - 40.7** | **37.7 BL,A,B** | **34.4 – 40.0** |
| **ALBUMIN SERUM CONCENTRATION** (g/L) | | | | | | | | |
| EARLY | **28.0** | **27.5 - 33.4** | **17.9 BL** | **17.0 - 19.1** | **16.8 BL** | **16.1 - 17.7** | **13.5 $,BL,A,B** | **10.8 - 16.4** |
| LATE | **29.0** | **28.4 - 30.9** | **17.3 *,BL** | **14.1 - 19.7** | **15.6 *$,BL,A** | **13.0 - 16.1** | **13.7 $,BL,A** | **11.4 - 15.4** |
| SEPSIS | **31.4** | **29.9 - 31.7** | **17.0 BL** | **16.3 - 19.1** | **18.5 $,BL** | **17.4 - 19.4** | **18.5 $,BL** | **18.1 - 19.7** |
| SHAM | **33.3** | **32.6 - 33.9** | **22.2 *,BL** | **21.9 - 23.3** | **18.5 *,BL,A** | **18.4 - 18.8** | **18.7 BL,A** | **16.1 - 20.7** |
| **SERUM GLUCOSE** (mmol/L) | | | | | | | | |
| EARLY | **4.8** | **4.4 - 6.1** | **4.5** | **4.0 - 4.7** | **7.2 $,BL,A** | **6.5 - 9.3** | **10.5 BL,A,B** | **7.1 - 14.7** |
| LATE | **6.4** | **4.8 - 6.8** | **4.0 BL** | **3.3 - 4.7** | **5.0 BL,A** | **3.8 - 6.1** | **12.9 *,BL,A,B** | **7.5 – 14.0** |
| SEPSIS | **5.2** | **5.0 - 7.3** | **3.9 BL** | **3.5 - 4.4** | **4.9 $** | **4.5 - 5.9** | **7.4 BL,A,B** | **6.7 - 11.3** |
| SHAM | 6.4 | 5.8 - 8.5 | 4.4 | 3.6 - 5.8 | 6.1 | 5.8 - 6.8 | **5.4 *** | **5.2 - 7.4** |
| **SERUM UREA CONCENTRATION** (mmol/L) | | | | | | | | |
| EARLY | **3.4** | **2.6 - 3.9** | **6.4 BL** | **5.0 - 7.9** | **7.6 BL,A** | **6.0 - 8.1** | **8.7 BL,A,B** | **6.6 - 9.3** |
| LATE | **3.1** | **2.8 - 3.7** | **7.7 BL** | **5.8 - 9.1** | **7.4 BL** | **6.9 - 11.5** | **8.5 BL** | **6.9 - 9.8** |
| SEPSIS | **3.2** | **2.6 – 4.0** | **6.5 BL** | **5.3 - 7.7** | **6.7 BL** | **6.0 - 7.2** | **8.7 BL,A,B** | **8.4 - 9.4** |
| SHAM | 3.6 | 2.9 - 5.1 | 4.8 | 4.1 - 5.7 | 4.3 | 4.0 – 7.0 | 5.3 | 3.9 - 7.5 |
| **SERUM CREATININE CONCENTRATION** (μmol/L) | | | | | | | | |
| EARLY | **103** | **72 - 110** | **95** | **90 – 119** | **130 BL,A** | **104 - 148** | **208 BL,A,B** | **181 - 238** |
| LATE | **92** | **78 - 100** | 116 | 98 – 207 | **172 BL** | **126 - 304** | **192 BL** | **177 - 261** |
| SEPSIS | **83** | **81 – 97** | **97** | **76 – 127** | **103 BL,A** | **94 - 150** | **222 BL,A,B** | **209 - 266** |
| SHAM | 103 | 91 - 115 | 118 | 86 - 136 | 97 | 71 - 182 | 177 | 81 - 225 |
| **TUMOR NECROSIS FACTOR ALPHA (TNF-α)** (pg/L) | | | | | | | | |
| EARLY | 0.0240 | 0.0105 - 0.0240 | 0.0208 | 0.0162 - 0.0240 | 0.0240 | 0.0197 - 0.0402 | 0.0176 | 0.00778 - 0.0288 |
| LATE | 0.0240 | 0.0189 - 0.0325 | 0.0189 | 0.00928 - 0.0381 | 0.0695 | 0.0108 - 0.232 | 0.0240 | 0.0177 - 0.162 |
| SEPSIS | 0.0223 | 0.00221 - 0.0245 | 0.0256 | 0.0204 - 0.0386 | 0.0449 | 0.0250 - 0.0677 | 0.0231 | 0.0151 - 0.114 |
| SHAM | 0.0240 | 0.0240 - 0.430 | 0.0240 | 0.0219 - 0.230 | 0.0151 | 0.00608 - 0.0240 | 0.0240 | 0.0169 - 0.336 |
| **INTERLEUKIN 1 ALPHA** (pg/L) | | | | | | | | |
| EARLY | **0.0239** | **0.00547 - 0.0295** | **0.0347 BL** | **0.0161 - 0.0627** | **0.0467 BL,A** | **0.0318 - 0.124** | **0.102 BL,A,B** | **0.0845 - 0.207** |
| LATE | 0.00672 | 0.00439 - 0.0163 | 0.0164 | 0.0109 - 0.0507 | 0.0194 | 0.0103 - 0.129 | 0.0817 | 0.0198 - 0.166 |
| SEPSIS | **0.0106** | **0.00500 - 0.0173** | **0.0188** | **0.00981 - 0.0263** | **0.0285 BL,A** | **0.0229 - 0.0447** | **0.0585 BL,A,B** | **0.0565 - 0.0614** |
| SHAM | 0.00533 | 0.00343 - 0.00974 | 0.00999 | 0.00562 - 0.0189 | 0.0199 | 0.0123 - 0.0484 | 0.0188 | 0.0112 - 0.192 |
| **INTERLEUKIN 1 BETA** (pg/L) | | | | | | | | |
| EARLY | **0.0465** | **0.0418 - 0.0898** | **0.587 BL** | **0.503 - 1.472** | **0.926 BL,A** | **0.572 - 2.099** | **2.756 BL,A,B** | **1.791 - 4.484** |
| LATE | **0.0778** | **0.0419 - 0.122** | **0.656 BL** | **0.581 - 0.740** | **0.838 BL** | **0.580 - 3.610** | **1.200 BL,A** | **0.923 - 3.602** |
| SEPSIS | **0.0422** | **0.0262 - 0.113** | **0.688 BL** | **0.584 - 0.750** | **0.901 BL,A** | **0.868 - 0.960** | **2.904 BL,A,B** | **1.661 - 3.818** |
| SHAM | **0.0955** | **0.0548 - 0.122** | **0.0474** | **0.0369 - 0.399** | **0.249 BL,A** | **0.0965 - 0.838** | **0.582 BL,A,B** | **0.135 - 1.802** |
| **INTERLEUKIN-1 RECEPTOR ANTAGONIST** (pg/L) | | | | | | | | |
| EARLY | **0.816** | **0.369 - 2.612** | **41.795 BL** | **20.589 - 55.612** | **56.237 BL,A** | **29.135 - 80.640** | **57.691 BL,A** | **39.733 - 73.398** |
| LATE | **0.333** | **0.179 - 1.896** | **31.350 BL** | **23.157 - 43.645** | **51.806 BL** | **31.957 - 59.313** | **50.332 BL** | **41.777 - 60.158** |
| SEPSIS | **0.187** | **0.109 - 0.889** | **26.403 BL** | **23.469 - 32.238** | **37.905 BL** | **29.794 - 53.558** | **41.508 BL** | **27.127 - 80.249** |
| SHAM | 0.338 | 0.173 - 0.741 | 0.954 | 0.583 - 15.665 | 14.385 | 4.974 - 30.404 | 33.078 | 21.633 - 49.682 |
| **INTERLEUKIN 2** (pg/L) | | | | | | | | |
| EARLY | **0.0383** | **0.00373 - 0.0775** | 0.0389 | 0.0141 - 0.122 | **0.0401 BL** | **0.0150 - 0.377** | **0.0361 BL** | **0.0295 - 0.482** |
| LATE | **0.0284** | **0.0134 - 0.0554** | **0.0518 BL** | **0.0342 - 0.137** | **0.0387 BL** | **0.0196 - 0.102** | **0.0327 BL** | **0.0239 - 0.128** |
| SEPSIS | 0.0282 | 0.0180 - 0.0455 | 0.0339 | 0.00402 - 0.0735 | 0.0491 | 0.00669 - 0.0851 | 0.0702 | 0.0112 - 0.135 |
| SHAM | 0.0120 | 0.00838 - 0.0231 | 0.0113 | 0.00645 - 0.0322 | 0.0326 | 0.0184 - 0.0422 | 0.0267 | 0.00540 - 0.0294 |
| **INTERLEUKIN 6** (pg/L) | | | | | | | | |
| EARLY | **0.0403** | **0.0292 - 0.0537** | **2.015 BL** | **1.505 - 2.964** | **3.200 BL,A** | **2.002 - 4.304** | **4.963 BL,A,B** | **4.134 - 7.122** |
| LATE | **0.0294** | **0.0178 - 0.0385** | **1.878 BL** | **1.439 - 2.195** | **2.769 BL,A** | **1.962 - 4.189** | **4.312 BL,A** | **3.116 - 6.100** |
| SEPSIS | **0.0269** | **0.0240 - 0.0373** | **1.881 BL** | **1.256 - 2.407** | **2.541 BL,A** | **2.369 - 3.542** | **5.009 BL,A,B** | **3.834 - 5.975** |
| SHAM | **0.0223** | **0.0191 - 0.0328** | **0.282 BL** | **0.0815 - 1.894** | **1.982 BL,A** | **0.746 - 3.053** | **3.309 BL,A,B** | **0.436 - 4.661** |
| **INTERLEUKIN 8** (pg/L) | | | | | | | | |
| EARLY | **0.0120** | **0.00777 - 0.0175** | **0.259 BL** | **0.130 - 0.334** | **0.552 BL,A** | **0.207 - 0.675** | **0.708 BL,A,B** | **0.437 - 4.301** |
| LATE | **0.0126** | **0.0120 - 0.0267** | **0.355 BL** | **0.125 - 0.444** | **0.703 BL,A** | **0.261 - 3.571** | **1.087 BL,A,B** | **0.849 - 2.965** |
| SEPSIS | **0.00754 *** | **0.00255 - 0.0117** | **0.241 BL** | **0.220 - 0.319** | **0.437 BL,A** | **0.287 - 0.744** | **1.694 BL,A,B** | **0.463 - 2.884** |
| SHAM | **0.0357 *** | **0.0120 - 0.204** | **0.0228 BL** | **0.0113 - 0.513** | **0.110 BL,A** | **0.0184 - 0.740** | **0.522 BL,A,B** | **0.371 - 1.958** |
| **INTERLEUKIN 10** (pg/L) | | | | | | | | |
| EARLY | **0.356** | **0.0765 - 0.418** | **0.682 *,BL** | **0.362 - 1.319** | **0.541 BL** | **0.308 - 2.571** | **0.900 *,BL,A,B** | **0.540 - 3.732** |
| LATE | **0.117** | **0.0669 - 0.341** | **0.492 BL** | **0.286 - 0.542** | **0.481 BL** | **0.374 - 0.738** | **0.571 BL** | **0.385 - 2.158** |
| SEPSIS | **0.0835** | **0.0478 - 0.356** | **0.350** | **0.296 - 0.474** | **0.519 BL** | **0.318 - 0.528** | **0.756 BL,A,B** | **0.566 - 1.092** |
| SHAM | 0.0522 | 0.0418 - 0.150 | **0.162 *** | **0.0594 - 0.253** | 0.174 | 0.0740 - 0.257 | **0.174 *** | **0.0688 - 0.249** |
| Legend: **bold** layout – statistical significance; ***** - significant difference compared to the SHAM group (Kruskall Wallis ANOVA); **$** - intergroup significant difference towards SEPSIS group (Kruskall Wallis ANOVA); **BL** – significant difference towards BASELINE measurement (Friedman ANOVA on repeated measurements); **A** - significant difference towards TP-A measurement (Friedman ANOVA on repeated measurements); **B** - significant difference towards TP-B measurement (Friedman ANOVA on repeated measurements) | | | | | | | | |

| **Table S2 – Hemodynamic, hematologic, laboratory parameters and cytokine levels (septic animals exposed to HA versus septic animals without HA)** | | | | | | | | |
| --- | --- | --- | --- | --- | --- | --- | --- | --- |
|  | **TP -0** | | **TP-A** | | **TP-B** | | **TP-D** | |
|  | **Median** | **25 – 75**  **percentile** | **Median** | **25 – 75**  **percentile** | **Median** | **25 – 75**  **percentile** | **Median** | **25 – 75**  **percentile** |
| **HEART RATE** (beats/minute) | | | | | | | | |
| SEP-HA | **94** | **86 - 104** | **133 BL** | **106 - 155** | **196 BL.A** | **165 - 226** | **190 *.BL.A** | **171 - 213** |
| SEPSIS | **93** | **84 - 109** | **119** | **82 - 142** | **210 BL.A** | **156 – 219** | **268 BL.A** | **244 - 283** |
| **MEAN ARTERIAL PRESSURE** (mmHg) | | | | | | | | |
| SEP-HA | **88** | **72 - 91** | **64 BL** | **63 - 65** | **66 BL** | **58 - 71** | **58 BL.A.B** | **41 - 63** |
| SEPSIS | **74** | **66 - 87** | **63 BL** | **60 - 66** | **71 A** | **65 - 71** | **58 BL.A.B** | **51 - 60** |
| **MEAN PULMONARY ARTERIAL PRESSURE** (mmHg) | | | | | | | | |
| SEP-HA | **18** | **16 - 21** | **25 BL** | **23 - 29** | **29 BL** | **24 - 34** | **34 BL** | **28 - 40** |
| SEPSIS | **18** | **16 - 20** | **28 BL** | **21 - 29** | **22** | **18 - 24** | **36 BL** | **25 - 43** |
| **CENTRAL VENOUS PRESSURE** (mmHg) | | | | | | | | |
| SEP-HA | **10** | **8 - 11** | **16 BL** | **15 - 18** | **15 BL** | **13 - 17** | **15 *.BL.A** | **13 - 16** |
| SEPSIS | **10** | **9 - 14** | **20 BL** | **15 - 21** | **18 BL** | **15 - 20** | **19 *.BL** | **18 - 21** |
| **PULMONARY ARTERIAL OCCLUDED PRESSURE** (mmHg) | | | | | | | | |
| SEP-HA | **9** | **7 - 9** | **12 BL** | **10 - 13** | **11 BL.A** | **10 - 12** | **12 BL.B** | **11 - 17** |
| SEPSIS | **8** | **7 - 9** | **12 BL** | **12 - 14** | **13 BL** | **11 - 13** | **16 BL.A.B** | **13 - 18** |
| **CARDIAC OUTPUT** (l/min) | | | | | | | | |
| SEP-HA | **3.1** | **3.0 - 3.5** | **4.3 BL** | **3.5 - 5.3** | **4.2 *.BL** | **3.3 - 4.6** | **2.0 *.BL.A.B** | **1.8 - 2.9** |
| SEPSIS | **3.5** | **2.8 - 3.6** | 4.8 | 3.4 - 6.0 | **7.1 *.BL** | **5.5 - 8.8** | **6.1 *.BL** | **3.6 - 6.7** |
| **WEIGHTED CARDIAC OUTPUT** (ml/min/kg) | | | | | | | | |
| SEP-HA | **67** | **61 - 71** | **93 BL** | **68 - 108** | **80 *.BL** | **65 - 90** | **39 *.BL.A.B** | **34 - 55** |
| SEPSIS | **59** | **56 - 68** | 95 | 72 - 103 | **134 *.BL** | **107 - 168** | **109 *.BL** | **75 - 114** |
| **STROKE VOLUME** (ml) | | | | | | | | |
| SEP-HA | **35** | **30 - 47** | **33 BL** | **31 - 40** | **21 *.BL** | **15 - 24** | **10 *.BL.A.B** | **9 - 15** |
| SEPSIS | 38 | 29- 40 | 40 | 38 - 45 | **35 *.BL** | **34 - 40** | **23 *.BL** | **14 - 28** |
| **SYSTEMIC VASCULAR RESISTANCE** (dyn·s/cm^5^) | | | | | | | | |
| SEP-HA | **1800** | **1503 - 1975** | **857 BL** | **673 - 1216** | **1013 *.BL** | **892 - 1283** | **1250 *.BL.A.B** | **1156 - 1466** |
| SEPSIS | **1723** | **1205 - 2062** | **730** | 678 - 990 | **586 *.BL** | **411 - 827** | **525 *.BL** | **514 - 879** |
| **PULMONARY VASCULAR RESISTANCE** (dyn·s/cm^5^) | | | | | | | | |
| SEP-HA | **422** | **390 - 527** | **244 BL** | **171 - 377** | **376 *** | **267 - 566** | **767 A.B** | **469 - 1066** |
| SEPSIS | 400 | 343 - 482 | 212 | 153 - 319 | **90 *** | **56 - 196** | 246 | 151 - 341 |
| **GLOBAL END-DIASTOLIC VOLUME INDEX** (ml/kg) | | | | | | | | |
| SEP-HA | **10.4** | **9.8 - 11.0** | **9.3 BL** | **8.4 - 10.2** | **7.8 *, BL, A** | **6.0 – 9.0** | 5.8 | 5.2 - 6.4 |
| SEPSIS | 10.0 | 9.6 - 10.2 | 10.5 | 9.3 – 11.4 | **9.4 *** | **8.9 - 10.5** | 9.0 | 8.4 - 9.5 |
| **EXTRA-VASCULAR LUNG WATER INDEX** (ml/kg) | | | | | | | | |
| SEP-HA | 7.9 | 7.2 - 9.3 | 8.4 | 8.0 - 9.2 | 7.5 | 6.8 – 8.0 | 19.6 | 10.2 - 28.9 |
| SEPSIS | 7.4 | 7.1 - 8.1 | 8.9 | 8.5 - 9.5 | 8.4 | 7.3 - 9.4 | 8.5 | 7.5 - 9.4 |
| **PULMONARY VASCULATURE PERMEABILITY INDEX** | | | | | | | | |
| SEP-HA | **3.1** | **2.7 - 3.2** | **3.6 BL** | **3.3 - 3.9** | **3.8 BL** | **3.4 - 5.5** | 7.9 | 7.9 - 7.90 |
| SEPSIS | 3.1 | 2.9 - 3.5 | 3.5 | 3.2 - 3.7 | 3.2 | 3.1 - 4.1 | 3.8 | 3.5 – 4.0 |
| **LEFT VENTRICULAR CONTRACTILITY (dPmax)** (mmHg/s) | | | | | | | | |
| SEP-HA | **753** | **611 - 831** | **840** | **644 - 1009** | **2225 BL,A** | **1614 - 2484** | **2705 BL,A,B** | **2489 - 2872** |
| SEPSIS | 860 | 496 - 913 | **725** | **570 - 755** | **1360 A** | **1235 - 1647** | **1922 A** | **1144 - 2700** |
| **CARDIAC FUNCTION INDEX** (unit per minute) | | | | | | | | |
| SEP-HA | 6.6 | 5.8 - 7.6 | 11.0 | 7.9 – 12.0 | 12.0 | 2.8 - 14.5 | 7.6 | 6.9 - 8.2 |
| SEPSIS | **6.9** | **6.2 - 7.7** | **11.0** | **6.8 – 12.0** | **11.6 BL,A** | **9.2 – 14.0** | 14.5 | 14.0 – 15.0 |
| **PULSE PRESSURE VARIATION** (%) | | | | | | | | |
| SEP-HA | **12** | **9 - 14** | 17 | 14 - 20 | **28 *,BL** | **19 – 30** | **20 BL** | **10 - 27** |
| SEPSIS | **12** | **8 - 13** | **12** | **10 - 21** | **17 *,BL,A** | **13 - 22** | **26 BL** | **20 - 32** |
| **SERUM LACTATE LEVEL** (mmol/L) | | | | | | | | |
| SEP-HA | **1.1** | **1.0 - 1.2** | **1.3** | **1.1 - 1.6** | **3.7 BL,A** | **2.4 - 4.4** | **11.9 BL,A,B** | **8.0 - 16.2** |
| SEPSIS | **1.3** | **1.0 - 1.7** | **1.1** | **1.0 - 1.2** | **2.1 A** | **1.8 - 2.9** | **8.6 BL,A,B** | **6.6 - 11.6** |
| **MIXED VENOUS OXYGEN SATURATION (S_V_O_2_)** (%) | | | | | | | | |
| SEP-HA | **57.4** | **49.9 - 61.5** | **56.9** | **51.6 - 63.7** | 59.6 | 51.3 – 64.0 | **35.7 BL,A** | **30.7 - 43.4** |
| SEPSIS | 54.6 | 46.5 - 63.0 | 57.0 | 28.0 - 60.6 | 58.9 | 55.2 - 61.6 | 33.3 | 30.0 - 36.6 |
| **HOROWITZ INDEX (PaO_2_/FiO_2_)** (mmHg) | | | | | | | | |
| SEP-HA | **473** | **433 - 513** | **410 BL** | **350 - 451** | **352 BL,A** | **303 - 454** | **361 BL** | **307 - 412** |
| SEPSIS | **470** | **456 - 477** | **430 BL** | **376 - 457** | **370 BL** | **344 - 404** | **385 BL** | **356 – 434** |
| **ARTERIAL pH** | | | | | | | | |
| SEP-HA | **7.52** | **7.50 - 7.57** | **7.40 BL** | **7.38 - 7.42** | **7.29 BL,A** | **7.16 - 7.29** | **6.98 BL,A,B** | **6.87 - 7.02** |
| SEPSIS | **7.54** | **7.51 - 7.56** | **7.42 BL** | **7.40 - 7.46** | **7.34 BL,A** | **7.26 - 7.38** | **7.03 BL,A,B** | **6.98 - 7.09** |
| **ARTERIAL BASE EXCESS** (mmol/L) | | | | | | | | |
| SEP-HA | **6.8** | **5.0 - 8.4** | **-0.2 *,BL** | **-1.3 - 0.9** | **-5.8 *,BL,A** | **-10.6 - -4.3** | **-21.4 BL,A,B** | **-25.1 - -19.2** |
| SEPSIS | **6.2** | **4.2 - 8.2** | **1.8 *** | **1.1 - 2.5** | **-1.0 *,BL,A** | **-2.5 - -0.7** | **-16.9 BL,A,B** | **-18.5 - -13.2** |
| **HEMOGLOBIN CONCENTRATION** (g/dL) | | | | | | | | |
| SEP-HA | **101** | **89 - 106** | **113 BL** | **106 - 122** | **128 BL,A** | **121 - 137** | **132 *,BL,A** | **120 - 139** |
| SEPSIS | **101** | **95 - 103** | **102** | **97 – 111** | **122 BL,A** | **117 - 127** | **145 *,BL,A,B** | **139 - 152** |
| **LEUKOCYTE COUNT** (10^9^/L) | | | | | | | | |
| SEP-HA | **17.0** | **14.6 - 19.1** | **7.7 BL** | **3.6 – 9.0** | **7.0 BL** | **4.4 - 8.6** | **6.1 BL** | **2.0 - 8.9** |
| SEPSIS | **13.6** | **13.1 - 16.7** | **5.9 BL** | **4.4 - 7.7** | **3.5 BL** | **3.1 - 6.2** | **4.0 BL,A** | **2.2 - 6.8** |
| **PLATELET COUNT** (10^9^/L) | | | | | | | | |
| SEP-HA | **357** | **322 - 422** | **160 BL** | **123 - 178** | **81 BL,A** | **63 - 106** | **66 *,BL,A,B** | **51 - 90** |
| SEPSIS | **324** | **297 - 367** | **148 BL** | **125 – 187** | **112 BL,A** | **101 – 121** | **24 *,BL,A,B** | **11 - 54** |
| **SERUM PROTEIN CONCENTRATION** (g/L) | | | | | | | | |
| SEP-HA | **55.1** | **51.3 - 59.7** | **33.8 BL** | **31.6 - 35.7** | **35.4 BL** | **32.6 - 38.2** | **33.1 BL** | **29.1 - 37.1** |
| SEPSIS | **52.6** | **48.6 - 54.1** | **30.4,BL** | **28.7 – 32.0** | **34.7 BL,A** | **32.5 - 37.2** | **39.1 BL,A** | **34.0 - 41.4** |
| **ALBUMIN SERUM CONCENTRATION** (g/L) | | | | | | | | |
| SEP-HA | **28.9** | **27.8 - 32.8** | **17.8 BL** | **16.5 - 19.3** | **16.0 *,BL,A** | **15.7 - 17.6** | **13.5 *,BL,A,B** | **11.1 - 15.8** |
| SEPSIS | **31.4** | **29.9 - 31.7** | **17.0 BL** | **16.3 - 19.1** | **18.5 *,BL** | **17.4 - 19.4** | **18.5 *,BL** | **18.1 - 19.7** |
| **SERUM GLUCOSE** (mmol/L) | | | | | | | | |
| SEP-HA | **5.6** | **4.4 - 6.7** | **4.2 BL** | **3.5 - 4.7** | **6.5 *,BL,A** | **5.0 - 9.2** | **12.9 BL,A,B** | **7.4 - 14.7** |
| SEPSIS | **5.2** | **5.0 - 7.3** | **3.9 BL** | **3.5 - 4.4** | **4.9 *** | **4.5 - 5.9** | **7.4 BL,A,B** | **6.7 - 11.3** |
| **SERUM UREA CONCENTRATION** (mmol/L) | | | | | | | | |
| SEP-HA | **3.3** | **2.7 - 3.8** | **7.3 BL** | **5.3 - 8.3** | **7.5 BL,A** | **6.4 - 9.7** | **8.5 BL,A,B** | **6.7 - 9.5** |
| SEPSIS | **3.2** | **2.6 – 4.0** | **6.5 BL** | **5.3 - 7.7** | **6.7 BL** | **6.0 - 7.2** | **8.7 BL,A,B** | **8.4 - 9.4** |
| **SERUM CREATININE CONCENTRATION** (μmol/L) | | | | | | | | |
| SEP-HA | **92** | **75 - 103** | **106** | **95 - 130** | **131 BL,A** | **121 - 204** | **204 BL,A,B** | **177 - 245** |
| SEPSIS | **83** | **81 – 97** | **97** | **76 – 127** | **103 BL,A** | **94 - 150** | **222 BL,A,B** | **209 - 266** |
| **TUMOR NECROSIS FACTOR ALPHA (TNF-α)** (pg/L) | | | | | | | | |
| SEP-HA | 0.0240 | 0.0163 - 0.0240 | 0.0208 | 0.0126 - 0.0275 | 0.0310 | 0.0152 - 0.0668 | 0.0185 | 0.0129 - 0.0483 |
| SEPSIS | 0.0223 | 0.00221 - 0.0245 | 0.0256 | 0.0204 - 0.0386 | 0.0449 | 0.0250 - 0.0677 | 0.0231 | 0.0151 - 0.114 |
| **INTERLEUKIN 1 ALPHA** (pg/L) | | | | | | | | |
| SEP-HA | 0.00810 | 0.00505 - 0.0263 | 0.0277 | 0.0116 - 0.0590 | 0.0382 | 0.0180 - 0.107 | 0.0955 | 0.0560 - 0.200 |
| SEPSIS | **0.0106** | **0.00500 - 0.0173** | **0.0188** | **0.00981 - 0.0263** | **0.0285 BL,A** | **0.0229 - 0.0447** | **0.0585 BL,A,B** | **0.0565 - 0.0614** |
| **INTERLEUKIN 1 BETA** (pg/L) | | | | | | | | |
| SEP-HA | **0.0768** | **0.0418 - 0.115** | **0.651 BL** | **0.521 - 0.827** | **0.882 BL,A** | **0.569 - 2.078** | **1.855 BL,A,B** | **1.196 - 4.350** |
| SEPSIS | **0.0422** | **0.0262 - 0.113** | **0.688 BL** | **0.584 - 0.750** | **0.901 BL,A** | **0.868 - 0.960** | **2.904 BL,A,B** | **1.661 - 3.818** |
| **INTERLEUKIN-1 RECEPTOR ANTAGONIST** (pg/L) | | | | | | | | |
| SEP-HA | **0.643** | **0.277 - 2.556** | **33.709 BL** | **21.639 - 51.365** | **53.628 BL** | **29.998 - 67.267** | **53.573 BL,A** | **40.407 - 68.726** |
| SEPSIS | **0.187** | **0.109 - 0.889** | **26.403 BL** | **23.469 - 32.238** | **37.905 BL** | **29.794 - 53.558** | **41.508 BL** | **27.127 - 80.249** |
| **INTERLEUKIN 2** (pg/L) | | | | | | | | |
| SEP-HA | **0.0327** | **0.00497 - 0.0633** | **0.0395 BL** | **0.0192 - 0.131** | **0.0394 BL** | **0.0169 - 0.187** | **0.0342 BL** | **0.0273 - 0.362** |
| SEPSIS | 0.0282 | 0.0180 - 0.0455 | 0.0339 | 0.00402 - 0.0735 | 0.0491 | 0.00669 - 0.0851 | 0.0702 | 0.0112 - 0.135 |
| **INTERLEUKIN 6** (pg/L) | | | | | | | | |
| SEP-HA | **0.0325** | **0.0247 - 0.0484** | **1.996 BL** | **1.421 - 2.393** | **3.167 BL,A** | **1.907 - 3.968** | **4.682 BL,A,B** | **3.717 - 6.436** |
| SEPSIS | **0.0269** | **0.0240 - 0.0373** | **1.881 BL** | **1.256 - 2.407** | **2.541 BL,A** | **2.369 - 3.542** | **5.009 BL,A,B** | **3.834 - 5.975** |
| **INTERLEUKIN 8** (pg/L) | | | | | | | | |
| SEP-HA | **0.0120 *** | **0.0120 - 0.0185** | **0.307 BL** | **0.119 - 0.414** | **0.612 BL,A** | **0.221 - 1.122** | **1.010 BL,A,B** | **0.621 - 4.067** |
| SEPSIS | **0.00754 *** | **0.00255 - 0.0117** | **0.241 BL** | **0.220 - 0.319** | **0.437 BL,A** | **0.287 - 0.744** | **1.694 BL,A,B** | **0.463 - 2.884** |
| **INTERLEUKIN 10** (pg/L) | | | | | | | | |
| SEP-HA | **0.126** | **0.0749 - 0.392** | **0.504 BL** | **0.331 - 0.902** | **0.511 BL** | **0.311 - 1.085** | **0.819 BL,A,B** | **0.447 - 2.201** |
| SEPSIS | **0.0835** | **0.0478 - 0.356** | **0.350** | **0.296 - 0.474** | **0.519** | **0.318 - 0.528** | **0.756** | **0.566 - 1.092** |
| Legend: **bold** layout – statistical signifikance; ***** - significant difference between groups (Mann Whitney test); **BL** – significant difference towards BASELINE measurement (Friedman ANOVA on repeated measurements); **A** - significant difference towards TP-A measurement (Friedman ANOVA on repeated measurements); **B** - significant difference towards TP-B measurement (Friedman ANOVA on repeated measurements)  Abbreviations: SEP-HA – combined results of septic groups exposed to hemoadsorption (both EARLY and LATE) | | | | | | | | |

| **Table S3 – Hemodynamic, hematologic, laboratory parameters and cytokine levels (sham operated animals exposed to HA versus historic controls)** | | | | | | | | |
| --- | --- | --- | --- | --- | --- | --- | --- | --- |
|  | **TP -0** | | **TP-A** | | **TP-B** | | **TP-D** | |
|  | **Median** | **25 - 75**  **percentile** | **Median** | **25 - 75 percentile** | **Median** | **25 - 75**  **percentile** | **Median** | **25 - 75**  **percentile** |
| **HEART RATE** (beats/minute) | | | | | | | | |
| SHAM | **103 *** | **99 - 112** | **63** | **61 - 100** | **116 *.A** | **109 - 186** | **179 BL.A** | **128 - 205** |
| CONTROL | **66 *** | **54 - 82** | 62 | 54 - 65 | **77 *** | **62 - 82** |  |  |
| **MEAN ARTERIAL PRESSURE** (mmHg) | | | | | | | | |
| SHAM | 81 | 72 - 87 | 64 | 61 - 66 | 65 | 64 - 66 | 62 | 54 - 66 |
| CONTROL | 71 | 63 - 77 | 69 | 68 - 71 | 71 | 67 - 75 |  |  |
| **MEAN PULMONARY ARTERIAL PRESSURE** (mmHg) | | | | | | | | |
| SHAM | **19** | **17 - 21** | **23 BL** | **20 - 32** | **33 BL** | **29 - 39** | **36 BL.A.B** | **26 - 38** |
| CONTROL | **18** | **16 - 22** | **24 BL** | **21 - 26** | **25 BL** | **23 - 27** |  |  |
| **CENTRAL VENOUS PRESSURE** (mmHg) | | | | | | | | |
| SHAM | **10** | **9 - 12** | **15 *.BL** | **15 - 18** | **16 *.BL.A** | **15 - 18** | **21 BL.A.B** | **17 - 22** |
| CONTROL | 12 | 10 - 13 | **12 *** | **11 - 13** | **12 *** | **11 - 14** |  |  |
| **PULMONARY ARTERIAL OCCLUDED PRESSURE** (mmHg) | | | | | | | | |
| SHAM | **8** | **7 - 9** | **13 BL** | **11 - 14** | **11 BL** | **9 - 13** | **10 BL** | **8 - 14** |
| CONTROL | **9** | **8 - 10** | **11 BL** | **11 - 13** | **13 BL** | **10 - 14** |  |  |
| **CARDIAC OUTPUT** (l/min) | | | | | | | | |
| SHAM | **3.2 *** | **3.0 - 3.7** | 2.6 | 2.6 - 3.7 | 4.9 | 3.5 - 5.2 | 3.9 | 3.3 - 5.1 |
| CONTROL | **2.8 *** | **2 - 2.9** | **3.1 BL** | **2.9 - 3.6** | **3.4 BL.A** | **3.3 - 4.1** |  |  |
| **WEIGHTED CARDIAC OUTPUT** (ml/min/kg) | | | | | | | | |
| SHAM | 63 | 56 - 75 | 51 | 45 - 77 | 86 | 75 - 89 | 77 | 56 - 100 |
| CONTROL | **57** | **50 - 76** | **75 BL** | **64 - 87** | **77 BL.A** | **73 - 105** |  |  |
| **STROKE VOLUME** (ml) | | | | | | | | |
| SHAM | 31 | 28 - 35 | 43 | 39 - 44 | **31 *** | **28 - 36** | 26 | 16 - 39 |
| CONTROL | **39** | **32 - 48** | **51 BL** | **44 - 61** | **49 *.BL.A** | **42 - 57** |  |  |
| **SYSTEMIC VASCULAR RESISTANCE** (dyn·s/cm^5^) | | | | | | | | |
| SHAM | **1929** | **1353 - 1995** | 1343 | 1105 - 1584 | **769 BL** | **713 - 1187** | **800 BL** | **646 - 1352** |
| CONTROL | **1937** | **1570 - 2185** | 1400 | 1336 - 1558 | **1333 BL** | **1095 - 1477** |  |  |
| **PULMONARY VASCULAR RESISTANCE** (dyn·s/cm^5^) | | | | | | | | |
| SHAM | **413 *** | **366 - 535** | 277 | 254 - 395 | **392 *** | **320 - 433** | 369 | 304 - 647 |
| CONTROL | **297 *** | **249 - 339** | 252 | 210 - 367 | **280 *** | **203 - 316** |  |  |
| **SERUM LACTATE LEVEL** (mmol/L) | | | | | | | | |
| SHAM | 1.1 | 1.0 - 1.2 | 1.1 | 0.8 - 1.7 | 1.7 | 1.2 - 1.8 | 3.4 | 1.7 - 5.9 |
| CONTROL | 1.1 | 1.0 - 1.4 | 1.0 | 1.0 – 1.0 | 1.0 | 1.0 – 1.0 |  |  |
| **MIXED VENOUS OXYGEN SATURATION (S_V_O_2_)** (%) | | | | | | | | |
| SHAM | **54.4** | **52.8 - 55.6** | **46.8 BL** | **39.3 - 52.6** | **63.5 A** | **45.7 - 68.5** | **60.0 A** | **42.4 - 64.4** |
| CONTROL | 47.7 | 41.3 - 54.6 | 43.6 | 40.6 - 46.3 | 49.1 | 42.4 - 54.2 |  |  |
| **NOREPINEPHRINE/NORADRENALINE RATE** (μg/kg/min) | | | | | | | | |
| SHAM | 0 | 0 - 0 | **0.140 *** | **0 - 0.243** | **0.480 *** | **0.365 - 1.572** | 2.920 | 1.018 - 10.640 |
| CONTROL | 0 | 0 - 0 | **0 *** | **0 - 0** | **0 *** | **0 - 0** |  |  |
| **HOROWITZ INDEX (PaO_2_/FiO_2_)** (mmHg) | | | | | | | | |
| SHAM | **479** | **474 - 507** | 445 | 243 - 486 | **352 BL** | **202 - 470** | **225 BL** | **193 - 461** |
| CONTROL | **479** | **443 - 516** | **344 BL** | **262 - 404** | **273 BL,A** | **232 - 344** |  |  |
| **ARTERIAL pH** | | | | | | | | |
| SHAM | **7.53** | **7.51 - 7.54** | **7.46** | **7.38 - 7.49** | **7.39 BL,A** | **7.34 - 7.44** | **7.30 *,BL,A,B** | **7.21 - 7.36** |
| CONTROL | **7.51** | **7.47 - 7.54** | 7.49 | 7.46 - 7.51 | **7.46 BL** | **7.41 - 7.47** |  |  |
| **ARTERIAL BASE EXCESS** (mmol/L) | | | | | | | | |
| SHAM | **5.3** | **4.5 - 7.2** | **2.1 BL** | **0.4 - 2.6** | **0.2 BL,A** | **-2.4 - 0.9** | **-2.9 * BL,A,B** | **-8.1 - 0.3** |
| CONTROL | **5.1** | **4.1 - 5.4** | 3.0 | 0.5 - 3.4 | **2.2 BL** | **-0.9 - 2.9** |  |  |
| **HEMOGLOBIN CONCENTRATION** (g/dL) | | | | | | | | |
| SHAM | **101 *** | **99 - 104** | **83** | **81 - 103** | **109 *,A** | **107 - 122** | **111 A** | **108 - 131** |
| CONTROL | **93 *** | **87 - 95** | **79 BL** | **74 - 84** | **82 *,BL** | **76 - 88** |  |  |
| **LEUKOCYTE COUNT** (10^9^/L) | | | | | | | | |
| SHAM | 15.3 | 12.4 - 16.3 | 12.9 | 9.0 - 14.6 | 8.5 | 5.8 - 14.9 | 3.1 | 2.6 - 14.9 |
| CONTROL | 19.1 | 17.3 - 21.3 | **7.5 BL** | **5.5 - 12.6** | **10.8 BL** | **8.0 - 12.2** |  |  |
| **PLATELET COUNT** (10^9^/L) | | | | | | | | |
| SHAM | **296** | **285 - 495** | **201 BL** | **167 - 313** | **141 BL,A** | **95 - 280** | **149 BL,A,B** | **77 - 262** |
| CONTROL | **285** | **226 - 315** | 236 | 213 - 271 | **241 BL** | **216 - 252** |  |  |
| **SERUM PROTEIN CONCENTRATION** (g/L) | | | | | | | | |
| SHAM | **54.9 *** | **52.8 - 58.1** | **40.0 *,BL** | **39.3 - 42.7** | **39.4 BL,A** | **37.2 - 40.7** | **37.7 BL,A,B** | **34.4 – 40.0** |
| CONTROL | **45.4 *** | **42.1 - 47.6** | **36.9 *,BL** | **36.5 - 39.3** | **37.6 BL,A** | **34.0 – 40.0** |  |  |
| **ALBUMIN SERUM CONCENTRATION** (g/L) | | | | | | | | |
| SHAM | **33.3 *** | **32.6 - 33.9** | **22.2 *,BL** | **21.9 - 23.3** | **18.5 BL,A** | **18.4 - 18.8** | **18.7 BL,A** | **16.1 - 20.7** |
| CONTROL | **24.2 *** | **22.4 – 28.0** | **19.0 *,BL** | **17.3 - 20.9** | **17.9 BL** | **17.2 - 20.2** |  |  |
| **SERUM GLUCOSE** (mmol/L) | | | | | | | | |
| SHAM | 6.4 | 5.8 - 8.5 | 4.4 | 3.6 - 5.8 | **6.1 *** | **5.8 - 6.8** | 5.4 | 5.2 - 7.4 |
| CONTROL | 5.9 | 4.7 - 6.6 | 4.2 | 3.8 - 4.6 | **4.1 *** | **3.4 - 4.6** |  |  |
| **SERUM UREA CONCENTRATION** (mmol/L) | | | | | | | | |
| SHAM | 3.6 | 2.9 - 5.1 | 4.8 | 4.1 - 5.7 | 4.3 | 4.0 – 7.0 | 5.3 | 3.9 - 7.5 |
| CONTROL | 5.0 | 4.2 - 5.5 | 5.0 | 3.0 - 5.6 | 4.4 | 3.2 - 4.6 |  |  |
| **SERUM CREATININE CONCENTRATION** (μmol/L) | | | | | | | | |
| SHAM | 103 | 91 - 115 | 118 | 86 - 136 | 97 | 71 - 182 | 177 | 81 - 225 |
| CONTROL | 95 | 80 - 103 | 88 | 77 - 102 | 85 | 68 - 93 |  |  |
| **TUMOR NECROSIS FACTOR ALPHA (TNF-α)** (pg/L) | | | | | | | | |
| SHAM | 0.0240 | 0.0240 - 0.430 | 0.0240 | 0.0219 - 0.230 | 0.0151 | 0.00608 - 0.0240 | 0.0240 | 0.0169 - 0.336 |
| CONTROL | 0.0500 | 0 - 0.100 | 0.0600 | 0.0534 - 0.0685 | 0.100 | 0 - 0.100 |  |  |
| **INTERLEUKIN 6** (pg/L) | | | | | | | | |
| SHAM | **0.0223** | **0.0191 - 0.0328** | **0.282** | **0.0815 - 1.894** | **1.982** | **0.746 - 3.053** | **3.309** | **0.436 - 4.661** |
| CONTROL | 0 | 0 - 0.100 | 0.0643 | 0.0482 - 0.212 | 0.500 | 0 - 1.500 |  |  |
| **INTERLEUKIN 8** (pg/L) | | | | | | | | |
| SHAM | **0.0357 *** | **0.0120 - 0.204** | **0.0228 BL** | **0.0113 - 0.513** | **0.110 BL,A** | **0.0184 - 0.740** | **0.522 BL,A,B** | **0.371 - 1.958** |
| CONTROL | **0 *** | **0 - 0** | **0.00574 *,BL** | **0.00486 - 0.0141** | **0 *,A** | **0 - 0** |  |  |
| Legend: **bold** layout – statistical signifikance; ***** - significant difference between groups (Mann Whitney test); **BL** – significant difference towards BASELINE measurement (Friedman ANOVA on repeated measurements); **A** - significant difference towards TP-A measurement (Friedman ANOVA on repeated measurements); **B** - significant difference towards TP-B measurement (Friedman ANOVA on repeated measurements) | | | | | | | | |
